# Recent Advances in Automated Mitosis Detection in Digital Pathology: A PRISMA-Guided Systematic Review with Evaluation-Regime Stratification (2018–2025)

**DOI:** 10.3390/biomedicines14061369

**Published:** 2026-06-17

**Authors:** Mohamed Albahri, Markus Kukuk, Felix Nensa, Georg Christian Lodde, Elisabeth Livingstone, Dirk Schadendorf

**Affiliations:** 1 Department of Computer Science, University of Applied Sciences Dortmund, 44227 Dortmund, Germany; 2 Institute for AI in Medicine (IKIM), University Hospital Essen, 45131 Essen, Germany; 3 Department of Dermatology, University Hospital Essen, 45147 Essen, Germanyelisabeth.livingstone@uk-essen.de (E.L.);

**Keywords:** mitosis detection, digital pathology, artificial intelligence, histopathology, precision oncology, systematic review

## Abstract

**Background/Objectives**: Recent advances in automated mitosis detection in H&E histopathology have expanded AI applications in digital pathology for tumor grading and proliferation assessment. However, reported performance remains difficult to interpret because it is strongly influenced by benchmark selection and heterogeneous evaluation regimes. This review examined how recent methodological advances, dataset context, and evaluation-regime stratification shape performance interpretation. **Methods**: We conducted a systematic review of peer-reviewed English-language studies published between January 2018 and December 2025. PubMed, Scopus, and IEEE Xplore were searched for mitosis detection, localization, or counting in H&E histopathology. After screening and full-text assessment, 66 studies met the inclusion criteria. We synthesized 60 method papers and considered 6 dataset/challenge descriptor papers separately. Extracted data included task formulation, datasets, evaluation regime, and outcomes. **Results**: The 60 method papers showed a methodological shift from patch/cell-level classification toward one-stage and two-stage detectors, dense segmentation/heatmap approaches, hybrid pipelines, and emerging robustness-oriented methods. F1 was reported in 59/60 studies, but evaluation practice was heterogeneous: custom hold-out testing predominated, whereas external validation and explicit domain-generalization protocols were uncommon. Evidence remained concentrated in legacy breast benchmarks, while MIDOG-family datasets anchored most robustness-oriented studies. Importantly, dataset names alone were insufficient to determine comparability; for example, “testing on ICPR2014” could refer to organizer-governed hidden-test scoring, post-challenge labels, or author-defined splits of public data. **Conclusions**: Automated mitosis detection research has diversified rapidly, but cross-study comparability remains limited by inconsistent evaluation and scarce cross-domain testing. Clearer reporting of dataset partitions, evaluation governance, and metrics, with more routine external or domain-held-out evaluation, would strengthen evidence for AI-driven digital pathology and precision oncology.

## 1. Introduction

Mitotic figure quantification in hematoxylin and eosin (H&E)–stained histopathology is a key component of tumor grading and prognostication in several cancer types, particularly breast cancer, where mitotic activity is used as a proxy for proliferative potential [1]. In routine practice, mitotic counting is performed manually—either at the microscope or digitally on whole-slide images (WSIs)—requiring assessment of multiple high-power fields (HPFs) and careful discrimination between true mitoses and visually similar “hard negative” structures (e.g., apoptotic bodies, pyknotic nuclei, crush/staining artifacts) [2]. This workflow is time-consuming and challenging due to tumor heterogeneity, severe class imbalance (mitoses are rare), and intra-/inter-observer variability, motivating computational methods that can improve consistency, reduce workload, and support more standardized proliferation assessment [3,4]. The clinical workflow has simultaneously shifted from purely microscope-based examination toward whole slide imaging (WSI), enabling algorithmic analysis at scale and establishing automated mitosis detection as a long-standing benchmark problem in computational pathology.

### 1.1. Evolution of Computational Paradigms

Early algorithmic systems were predominantly feature-engineered pipelines. These methods typically followed a multi-stage design—candidate proposal, handcrafted feature extraction (color, texture, morphology), and classification with conventional learners (e.g., SVMs)—and were often accompanied by stain handling and nuclei-focused preprocessing [5,6,7,8]. In their review, Jian et al. synthesized this classical view by organizing breast mitotic assessment into sequential processing steps (color normalization → nuclei detection/segmentation → feature extraction → classification) and by summarizing the public datasets that anchored early comparisons [9]. This pipeline framing clarified the methodological building blocks and the practical motivations behind preprocessing-heavy designs, but also highlighted an inherent limitation: performance could depend strongly on domain-specific feature choices and thresholding decisions that do not necessarily transfer across laboratories and scanners.

The rise of deep learning fundamentally altered the field’s modeling assumptions by replacing manual feature engineering with learned representations. Dif and Elberrichi et al. synthesized the first major wave of deep-learning approaches up to 2019, documenting the transition from pixel-wise CNN classification toward patch-wise classifiers, fully convolutional networks (FCNs), and region-based detection families such as Faster R-CNN. Their review also consolidated the technical motifs that repeatedly appeared in this period—transfer learning from ImageNet-pretrained backbones, stain normalization to mitigate inter-laboratory variability, multi-scale learning to incorporate contextual cues, and cascade designs intended to suppress false positives—and emphasized persistent obstacles such as severe class imbalance, limited numbers of annotated mitoses, scanner variability, and the tendency to overfit small benchmark datasets (e.g., ICPR2012 [5], AMIDA13 [10]) [11].

Since 2018, methodological diversification has accelerated. The research landscape has broadened beyond patch-level CNN classification to include proposal-driven two-stage detectors, one-stage detectors, dense prediction approaches, and increasingly transformer-inspired detection paradigms [12,13,14,15]. In parallel, weak-label training regimes (e.g., centroid- or box-level supervision) and explicit domain adaptation/generalization strategies have become more prominent, reflecting the field’s growing recognition that robustness under domain shift is not a peripheral concern but a primary requirement for reliable deployment [16,17,18,19]. For clinical implementation, such robustness is essential because scanner-, staining-, laboratory-, and tissue-context differences can change false-positive and false-negative behavior, thereby affecting mitotic-count reproducibility, threshold stability, and the reliability of grading support across deployment sites.

### 1.2. Benchmark Expansion and the Shift Toward Robustness

Historically, the dominance of breast cancer in automated mitosis detection research was driven in part by the availability of publicly released datasets and challenges, which provided a common substrate for method development and comparison [5,10,20]. This breast-centric benchmark landscape remains influential, but the evaluation landscape has expanded in two important directions.

First, modern benchmarks increasingly encode multi-source heterogeneity. Whereas earlier datasets often reflected limited center/scanner diversity, newer challenge-style benchmarks—most notably MIDOG21 [21] and MIDOG22 [22]—introduced explicit domain variation (e.g., across scanners and institutions) and evaluation protocols that stress cross-domain robustness. This evolution has pushed the community from a primary focus on within-dataset optimization toward models intended to tolerate domain shifts that more closely resemble real-world variability.

Second, the diversity of evaluation designs across publications has become more pronounced precisely as methods have become more powerful. Studies now commonly report results under a wide range of regimes: organizer-governed official test protocols, custom hold-out partitions, cross-validation, external validation on unseen datasets, and scanner-held-out or cross-dataset generalization setups. These regimes are not interchangeable. They encode different assumptions about what constitutes “generalization,” and they frequently yield substantially different performance estimates even for the same methodological family.

### 1.3. Related Reviews and What They Enable

Multiple reviews have consolidated key aspects of the field, particularly in breast histopathology, reflecting both the clinical importance of mitotic count and the historical dominance of challenge-driven public benchmarks that have structured methodological development and performance comparison in the field. Beyond pipeline-oriented syntheses (Jian et al. [9]) and deep-learning–focused methodological consolidation (Dif and Elberrichi [11]), additional narrative reviews have continued to organize the space of approaches and practical issues. Pan et al. reviewed breast-focused mitosis detection techniques in H&E images, grouping methods into broad categories (traditional, deep learning, hybrid, and other) and emphasizing recurring challenges such as class imbalance [23]. Mathew et al. provided a decade-scale review of computational methods for automated mitosis detection, grounding motivation in the workload and variability of manual assessment and summarizing algorithmic progress within the breast-centric landscape [24].

In parallel, review methodology has expanded from narrative synthesis to bibliometric mapping. Tan et al. conducted a PRISMA-guided scientometric analysis spanning 2004–2024 and reported 61 included breast-focused articles, providing a panoramic view of research activity through quantitative analyses (e.g., temporal patterns, geographic and institutional networks, and journal/publisher distributions) alongside qualitative organization of methodological directions [25]. This kind of work is well-suited for identifying research trajectories and the structure of the publication landscape.

### 1.4. Why Study-Level Evidence Synthesis Remains Difficult

Despite the value of these prior reviews, several structural limitations persist that directly affect how confidently one can compare reported results across the literature.

Breast-centric scope: Most existing reviews are breast-focused by design. This is appropriate given NHG-driven motivation and benchmark availability, but it incompletely reflects the contemporary landscape in which mitosis detection is increasingly evaluated under multi-domain conditions (including scanner diversity and broader tissue contexts), where domain shift and generalization are central challenges [21,26].

Macro-level mapping versus evidence extraction: Many reviews emphasize methodological overviews or trend mapping. Even when they discuss metrics, they typically do not aim to produce a standardized, study-level evidence table that aligns (for each paper) the train/test datasets, data partitioning strategy, evaluation regime, and the directly reported outcomes under that regime.

Protocol heterogeneity as a primary confounder: The literature spans evaluation regimes that encode fundamentally different strengths of evidence. Results derived from a custom hold-out split, k-fold cross-validation, an official challenge test server, external validation, or scanner-held-out domain generalization cannot be treated as directly comparable without explicit stratification. In practice, the same dataset family (e.g., legacy breast benchmarks) may appear under multiple regimes across papers, and reported performance can vary substantially depending on whether evaluation is organizer-governed versus study-defined. Consequently, headline metrics (often F1) can be influenced by evaluation design.

Dataset heterogeneity and governance: Benchmarks differ not only in size, but also in scanner composition, annotation process (including the number of annotators and their agreement), and test-set governance (e.g., hidden test sets in challenges). A result obtained on a small single-center dataset is not commensurate with performance under multi-scanner, institution-diverse, or domain-held-out conditions.

### 1.5. Rationale and Contribution of the Present Systematic Review

Unlike prior narrative or scientometric reviews, this review extracts study-level evidence and stratifies results by evaluation regime and dataset governance. Without dataset- and protocol-aware interpretation, cross-paper comparisons risk conflating fundamentally different problem settings. A two-stage detector reporting F1 = 0.96 under a custom hold-out split on ICPR2014 cannot be directly compared with a dense segmentation model reporting F1 = 0.72 under official challenge testing, nor with a domain-generalized model reporting F1 = 0.82 under scanner-held-out DG. These results reflect fundamentally different strengths of evidence. Here, domain refers to the data-generating context, including institution, scanner, staining, cell or cancer type, and species (human versus canine). Although these robustness axes are distinct and ideally should be examined separately, we group them under the broader concept of domain shift to keep the review tractable. We do not assume these sources are equivalent; where reported, we record the dominant shift axis separately.

These considerations motivate a systematic review that is explicitly evaluation-stratified and dataset-aware. The key requirement is not simply to catalog architectures, but to align methodological claims with the evidence context in which they were generated. Accordingly, the present work synthesizes studies on automated mitosis detection published between 2018 and 2025 under a PRISMA-guided framework and performs structured extraction of study-level evidence, explicitly mapping each study to its train/test datasets, evaluation type, and reported outcomes.

By linking methodological families to benchmark context and evaluation regime, this review provides a structured evidence map of where empirical support is strong, where it remains sparse, and how robustness claims differ across testing conditions—supporting a more reliable identification of approaches that demonstrate reproducible performance under clinically relevant domain shifts.

## 2. Methods

### 2.1. Reporting Framework and Review Scope

This review was planned and reported in line with PRISMA guidance [27]. The systematic review was registered on the Open Science Framework [28] (OSF; registration DOI: 10.17605/OSF.IO/UWMYR). This OSF record represents a retrospective registration/archive and should not be interpreted as a prospective preregistration.

The objective was to identify, appraise, and synthesize peer-reviewed English-language studies published between January 2018 and December 2025 that evaluated automated mitotic figure detection in H&E histopathology, including workflows operating at the patch/HPF level and whole-slide image (WSI) level. We included algorithmic studies addressing mitosis localization/detection, candidate screening, and mitotic counting when these were supported by quantitative evaluation. In addition, we retained a small set of dataset/challenge descriptor papers when they defined benchmarks and evaluation protocols repeatedly used by the included algorithmic studies.

### 2.2. Information Sources

Three bibliographic databases were searched: PubMed, Scopus, and IEEE Xplore. The last search was performed on 31 January 2026. Searches were executed to capture four concepts: (i) mitosis/mitotic figure targets, (ii) detection/localization/counting tasks, (iii) digital pathology imaging contexts, including H&E, histopathology, HPF, and WSI, and (iv) computational approaches ranging from classical pipelines to deep learning and robustness-oriented methods, including weak supervision, domain shift, domain generalization, and benchmark/challenge terminology.

### 2.3. Search Strategy

A Boolean strategy was developed to maximize sensitivity for mitosis detection in digital pathology while restricting retrieval to relevant imaging and computational contexts. The database-specific queries, as executed, are provided in Supplementary Data S3. Conceptually, the strategy combined terms for the target population/task, including “mitosis” and “mitotic figure”; detection-related terms, including “detect*”, “localiz*”, and “count*”; imaging-context terms, including “H&E”, “histopathology”, “WSI”, and related HPF/whole-slide terminology; and computational-method terms, including “machine learning”, “deep learning”, “CNN”, “weak supervision”, “domain shift”, “domain generalization”, and benchmark keywords such as MIDOG, TUPAC, MITOS, and AMIDA.

Database yields before exclusions and deduplication were PubMed 107, Scopus 341, and IEEE Xplore 51, resulting in 499 records.

### 2.4. Eligibility Criteria

Studies were included if they: (i) investigated automated mitotic figure detection, localization, or counting in histopathology imagery; (ii) used H&E-stained images in patch/HPF and/or WSI settings; (iii) reported quantitative performance for the detection/classification task, such as F1-score, precision/recall, AP, detection rate, or accuracy for classification-only formulations; and (iv) were peer-reviewed research articles or full conference papers indexed in the searched databases and published in English between January 2018 and December 2025.

Studies were excluded if they were reviews, surveys, editorials, commentaries, case reports, abstracts without full empirical reporting, or non-peer-reviewed records; if mitosis detection was not the primary task; if images were not H&E histopathology; if the report was not in English; or if no empirical evaluation relevant to the detection/classification objective was provided.

The 2018–2025 window was selected to focus on the contemporary deep-learning era of automated mitosis detection, following earlier reviews that had already summarized classical and early CNN-based approaches. English-language restriction was applied for feasibility and reproducibility of extraction, but this may introduce language bias and is acknowledged as a limitation.

### 2.5. Study Selection Process

The literature search identified 499 records. Following an initial exclusion of reviews and case reports (n = 61), 438 records remained for further processing. Deduplication across databases removed 88 overlapping entries, resulting in 350 unique publications. The deduplicated dataset comprised records retrieved from PubMed (n = 91), Scopus (n = 219), and IEEE Xplore (n = 40).

Titles and abstracts of the 350 unique records were screened against the predefined eligibility criteria, yielding 78 articles for full-text assessment. During full-text evaluation, 14 studies were excluded due to scope mismatches, including non-mitosis target tasks, non-H&E imaging modalities, or insufficient reporting of evaluation protocols. Two additional studies were identified through backward citation screening. The final included corpus comprised 66 studies, including 60 studies on automated mitosis detection and 6 dataset or challenge descriptor publications.

### 2.6. Review Process and Reviewer Roles

Titles/abstracts, full texts, data extraction, and quality assessment were performed by one reviewer with an audit by a second reviewer. Records were screened according to predefined eligibility criteria. Uncertain cases were resolved by discussion with the second reviewer. Where reporting was insufficient to determine the evaluation regime or dataset partition, the item was coded as unclear rather than inferred.

### 2.7. Handling Dataset/Challenge Descriptor Papers

The search strategy retrieved both algorithmic studies and dataset/challenge descriptor papers. The latter were retained when they defined benchmarks, annotation protocols, scoring rules, or official evaluation governance repeatedly used by the included algorithmic studies.

These descriptor papers were handled as a separate category during synthesis. They were not incorporated into methodological family counts or performance comparisons. Instead, they were used to document benchmark characteristics, label definitions, and organizer-specified evaluation frameworks that contextualize the method-focused literature.

### 2.8. Data Extraction and Coding

A standardized extraction template was applied at the study level. Extracted variables included author–year identifier, domain/tissue, training dataset(s), test dataset(s), evaluation type(s), task formulation, method description, and best reported test-set result. Extraction was consolidated into a structured evidence table covering the 60 algorithmic studies. When a paper reported multiple evaluations across datasets or regimes, the evidence table preserved the dataset-specific evaluation descriptors as stated by the authors rather than collapsing them into a single summary entry.

#### 2.8.1. Evaluation-Type Classification

Evaluation type was coded according to the regime explicitly associated with the reported results. The predefined categories were: official split/challenge test, custom hold-out split, cross-validation, external validation, explicit domain generalization, and unclear/insufficiently specified.

Official split/challenge testing was defined as organizer-defined evaluation with fixed partitions and/or benchmark/challenge test protocols, including settings with hidden labels and official scoring. Custom hold-out testing referred to author-defined train/test splits within or across datasets that were not described as official organizer splits. Cross-validation included k-fold or repeated resampling evaluation. External validation referred to training on one named dataset family and testing on a different named dataset family. Explicit domain generalization was coded when at least one domain, such as scanner, laboratory, or dataset domain, was held out entirely from training by design and used only for testing. Studies were coded as unclear when reporting did not allow confident assignment to one of these regimes.

#### 2.8.2. Method Taxonomy

Each algorithmic study was assigned to one primary method family based on the dominant localization mechanism used to generate candidate mitosis locations. The predefined families were: two-stage region/proposal detectors, one-stage object detectors, dense segmentation or heatmap-based detection, candidate-based cascades, handcrafted/classical machine-learning pipelines, deep feature extraction or patch/cell-level classification, and representation-learning/domain-adaptation/domain-generalization approaches.

For hybrid pipelines, family assignment was based on the component responsible for the main localization step rather than downstream refinement alone. Detailed decision rules for family assignment are provided in Supplementary Data S5.

### 2.9. Quality Assessment

Risk of bias and applicability concerns were assessed for all 60 algorithmic studies using a modified QUADAS-2 framework [29] tailored to mitosis detection in digital pathology. The tool covered data selection and split integrity, index-test development and evaluation integrity, reference-standard quality, outcome reporting, and applicability to the intended detection setting. Each signaling question was rated as Yes, No, or Unclear, and domain-level judgments were derived using predefined rules.

Because the included studies were algorithmic benchmark studies rather than diagnostic accuracy studies embedded in a uniform clinical pathway, the modified QUADAS-2 framework was used to support structured quality appraisal rather than quantitative weighting or exclusion from synthesis.

### 2.10. Synthesis Approach

Given heterogeneity in datasets, split constructions, reporting granularity, and evaluation regimes, we conducted a structured qualitative synthesis rather than a quantitative meta-analysis, consistent with SWiM principles [30]. The primary synthesis artifact was the standardized evidence table summarizing, for each algorithmic study, the domain/tissue, training and test datasets, evaluation type(s), primary method family, task formulation, and best reported test-set outcome.

Because the F1-score was the dominant outcome but its operational definition and evaluation granularity varied across studies, performance was reported exactly as stated by the original authors. Direct narrative comparisons were restricted to aligned dataset families and evaluation regimes, such as organizer-defined testing, custom hold-out testing, external validation, or explicit domain generalization. No formal publication-bias analysis or certainty-of-evidence grading was performed because the review did not pool effect sizes and the included studies were heterogeneous in datasets, evaluation protocols, and outcome definitions.

## 3. Results

### 3.1. Study Selection (PRISMA Flow)

**Figure 1:**
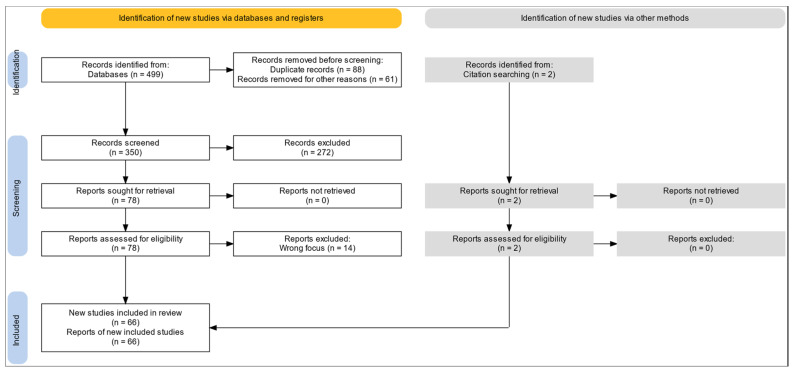
PRISMA study selection flow diagram [27]. Flow of record identification (databases and citation searching), duplicate removal, screening, full-text eligibility assessment, and final inclusion of studies in the systematic review.

The database search identified 499 records (PubMed: 107; Scopus: 341; IEEE Xplore: 51), as reported in the Methods. After removal of reviews and case reports (n = 61), 438 records remained. Duplicate removal excluded 88 records, yielding 350 unique publications for title/abstract screening. Seventy-eight studies underwent full-text assessment, of which 14 were excluded for wrong scope or insufficient evaluation reporting; two additional studies were identified via backward citation screening (Figure 1). In total, 66 studies met the inclusion criteria and were synthesized qualitatively, consistent with the predefined PRISMA-guided workflow described in Section 2.

### 3.2. Quality Assessment

**Figure 2:**
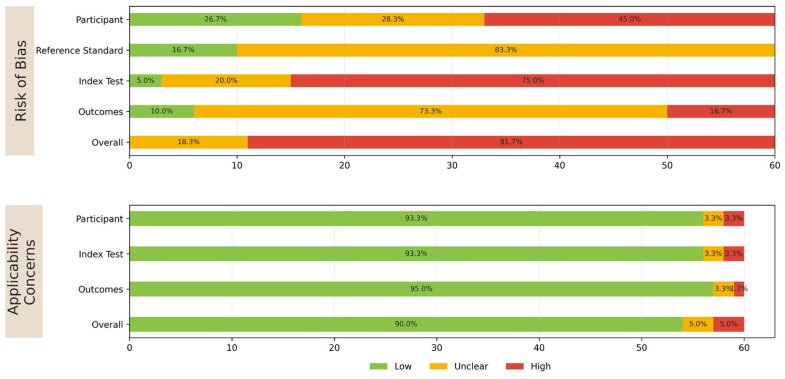
Summary stacked bar chart of the modified QUADAS-2 quality assessment across 60 included studies, showing the proportions of low, unclear, and high judgments for each risk-of-bias and applicability domain.

Risk of bias and applicability concerns for all 60 included algorithmic studies were assessed using our modified QUADAS-2 framework [29] (see Supplementary Data S4). Overall, the body of evidence showed substantial risk of bias despite generally low applicability concerns (Figure 2). Across all studies, the overall risk of bias was judged high in 83.3% and unclear in 16.7%, with no study rated low risk overall. The most problematic domain was Index Test, in which 76.7% of studies were rated high risk, followed by Participants (45.0% high risk). By contrast, Reference Standard (83.3% unclear) and Outcomes (73.3% unclear) were more often limited by insufficient reporting than by clearly high-risk methods.

Applicability concerns were considerably more favorable. Overall applicability concern was judged low in 91.7% of studies, with only 5.0% rated high concern. Low-concern judgments were also frequent across individual applicability domains, including Participants (95.0%), Index Test (95.0%), and Outcomes (96.7%). Taken together, these findings suggest that most studies addressed clinically relevant settings and outcomes, but the reliability of their reported performance was limited by weaknesses in study design, conduct, or reporting transparency.

### 3.3. Overview of Algorithmic Evidence and Evaluation Heterogeneity

**Figure 3:**
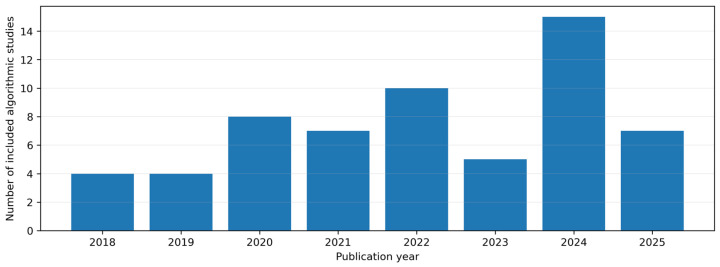
Included methodological studies by publication year (n = 60). Annual distribution of the 60 included methodological papers (2018–2025), highlighting growth over time with the highest volume in 2024 and sustained activity in 2025.

Across 60 included algorithmic studies (2018–2025) (Figure 3), the evidence base shows a clear temporal expansion and a parallel increase in evaluation heterogeneity. As summarized in Figure 3, annual output rose from a small set of early contributions in 2018–2019 (each 4/60, 6.7%) to a pronounced peak in 2024 (15/60, 25.0%), with sustained activity in 2022 (10/60, 16.7%) and continued publication in 2025 (7/60, 11.7%). Consistent with this growth, the included studies predominantly targeted object-level detection (53/60, 88.3%), with a smaller subset framing the problem as patch/cell-level classification (7/60, 11.7%). Most studies operated in breast histopathology (46/60, 76.7%), while multi-domain designs—explicitly spanning multiple domains/scanners/datasets—accounted for 12/60 (20.0%) (Table 1). The raw extracted study-level data underlying this overview are provided in Supplementary Data S1.

Methodologically, the corpus spans a broad set of detection paradigms (Table 1), with two-stage proposal-driven detectors and feature-centric pipelines remaining prominent alongside one-stage detectors and dense prediction approaches. Under the mutually exclusive primary taxonomy used for synthesis, two-stage R-CNN–family detectors represent 13/60 (21.7%), one-stage object detectors 11/60 (18.3%), dense segmentation/heatmap-based approaches 9/60 (15.0%), candidate cascades 5/60 (8.3%), handcrafted/classical ML pipelines 3/60 (5.0%), deep feature/patch-classification hybrids 12/60 (20.0%), and representation learning/DA–DG–oriented studies 7/60 (11.7%). Importantly, these families are not isolated from one another in practice: many pipelines are hybrid (e.g., detector → classifier, segmentation → classifier). To maintain consistent and transparent categorization, each study is assigned to the family that best reflects its primary localization mechanism rather than auxiliary refinement components (Table 1).

A central finding of this review is that evaluation design is a dominant source of heterogeneity, often exceeding architectural differences in its impact on interpretability and comparability. At the study level, custom hold-out evaluation was most common (42/60, 70.0%), whereas official split/challenge testing was reported by 24/60 (40.0%). Cross-validation appeared in 8/60 (13.3%), external validation in 9/60 (15.0%), and explicit domain generalization (DG) protocols in only 2/60 (3.3%). In 4/60 studies (6.7%), the evaluation regime was categorized as unclear/insufficiently specified, reflecting cases in which the reporting did not permit confident assignment to the predefined categories (Table 1). This protocol diversity is compounded by extensive multi-dataset reporting: based on dataset entries in the evidence table, 70.0% of studies reported results on ≥2 test datasets, and 61.7% reported ≥2 training datasets. Consequently, comparisons that do not stratify by evaluation type risk conflating within-domain performance estimates (often inflated under favorable hold-out designs) with more stringent organizer-scored, domain-held-out, or cross-dataset assessments.

Dataset usage further concentrates the evidence base around a small set of recurring public benchmarks while still exhibiting substantial breadth (Table 1). ICPR2014 [20] is the most frequently used test benchmark (37/60, 61.7%), followed by ICPR2012 [5] (25/60, 41.7%), TUPAC16 [74] (15/60, 25.0%), MIDOG21 [21] (11/60, 18.3%), AMIDA13 [10] (9/60, 15.0%), and MIDOG22 [22] (8/60, 13.3%), with additional evaluation on MIDOG++ [26] (3/60, 5.0%), GZMH [75] (3/60, 5.0%), and the veterinary WSI benchmarks MITOS_CMC [76] (2/60, 3.3%) and MITOS_CCMCT [77] (4/60, 6.7%). Notably, Table 2 makes explicit that the best reported F1 values are highly protocol contingent: upper bounds under custom hold-out and cross-validation can substantially exceed those observed under official/challenge, explicit DG, or external validation regimes. Table 2 also highlights two important nuances: (i) for several dataset–protocol combinations, the evidence base is sparse (e.g., DG and external validation on many datasets), and (ii) for ICPR2014, organizer-scored “official test” interpretation is not always verifiable from the included reports, warranting cautious handling of any purported official-test upper bounds and reinforcing the need for protocol-qualified synthesis.

Taken together, the overview synthesis and the protocol-stratified upper-bound summary (Table 2) support the core interpretive framework used throughout Section 3: methodological conclusions must be read jointly with evaluation regime and dataset context.

### 3.4. Dataset and Challenge Benchmark Papers Identified by the Review Search

**Figure 4:**
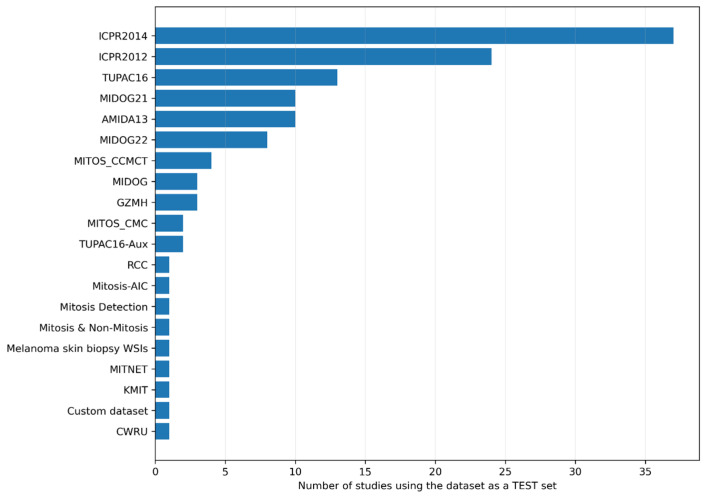
Test dataset usage frequency among methodological studies (n = 60). Number of methodological studies that used each dataset as a test set. Counts are not mutually exclusive because single studies may evaluate multiple test datasets.

Beyond algorithmic method papers, our PRISMA-guided search and screening also retrieved six dataset/challenge benchmark papers (TUPAC16 [74], MIDOG21 [21], MIDOG22 [22], MIDOG++ [26], MITOS_CMC [76], and MITOS_CCMCT [77]) that met the inclusion criteria as primary outputs of the database queries (rather than appearing only as evaluation datasets within methodological studies). These benchmark papers are included because they define data composition, annotation standards, and evaluation intent that underpin much of the algorithmic evidence synthesized in Section 5. A comprehensive inventory of all datasets mentioned in the included literature is provided in Supplementary Data S2 with brief summaries. Figure 4 summarizes the frequency of test dataset usage across included methodological studies.

### 3.5. Taxonomy of Methods Used

**Figure 5:**
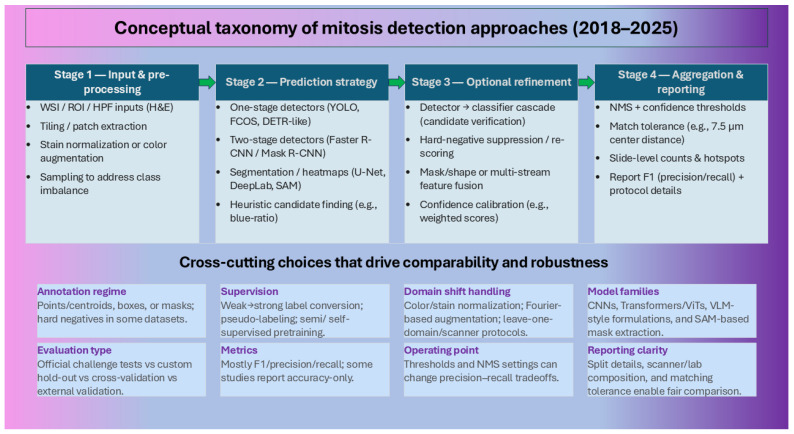
Conceptual taxonomy of automated mitosis detection workflows in H&E histopathology (2018–2025). The schematic summarizes recurring pipeline stages reported across the included studies: (Stage 1) input acquisition and pre-processing; (Stage 2) primary prediction strategy; (Stage 3) optional refinement to improve precision and localization; and (Stage 4) aggregation and reporting, including slide-/region-level outputs and performance evaluation.

**Figure 6:**
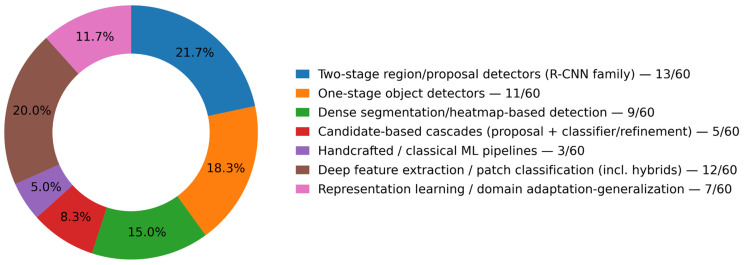
Methodological families according to the dominant detection paradigm reported in the Method taxonomy field.

Figure 5 presents a conceptual taxonomy of automated mitosis detection workflows in H&E histopathology reported between 2018 and 2025. Based on the extracted evidence table (2018–2025; n = 60) (Table 1), we organized included studies into seven methodological families according to the dominant detection paradigm reported in the “Method taxonomy” field. Because multiple pipelines were explicitly hybrid (e.g., segmentation → classification; detector → re-classification), categorization was anchored to the primary localization mechanism (Figure 6). In addition, we defined a separate robustness-focused family for studies whose method descriptions explicitly centered on representation learning and/or domain adaptation–generalization objectives (e.g., adversarial UDA, contrastive pretraining, Fourier-based DG), regardless of whether the downstream head was detector- or segmentation-based; this design choice affects where a small number of hybrid pipelines are discussed. Overall, proposal-based two-stage detectors and one-stage detectors remained the most frequent paradigms, while dense prediction, candidate cascades, and classical feature-engineered pipelines accounted for smaller shares of the evidence base.

#### 3.5.1. Two-Stage Region/Proposal Detectors (R-CNN Family; n = 13)

**Figure 7:**
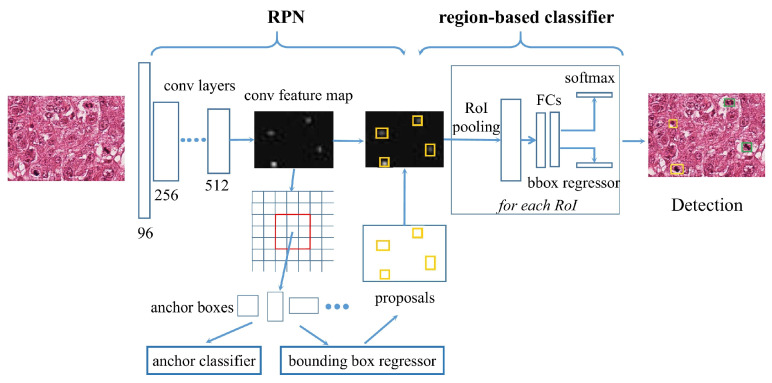
Example of two-stage region/proposal detectors (Li et al. [15]).

Thirteen studies operationalized mitosis detection as region proposal–driven small-object detection using Faster R-CNN/Mask R-CNN/Cascade R-CNN–type formulations, often augmented with downstream screening or re-classification stages. Early multi-stage formulations explicitly chained proposal-based detection with subsequent refinement and verification, exemplified by a pipeline that combined Faster R-CNN/region proposal network (RPN) detection with FCN-based box estimation and a final verification stage [15] (Figure 7). Subsequent work explored mask-driven candidate generation coupled to explicit feature fusion and secondary classifiers, including a two-stage design that used Mask R-CNN for high-recall candidate masks/boxes followed by a classifier combining hand-crafted mask-based features with deep features [34], and dedicated Mask R-CNN variants that jointly addressed detection and instance segmentation [83]. Architectural modifications to the core two-stage backbone were also common, including a modified Faster R-CNN “light head” configuration [13], and multistage pipelines that used Faster R-CNN for candidate detection followed by deep CNN refinement [14]. Several studies incorporated explicit proposal screening strategies designed to suppress false positives and handle hard negatives, such as spatial attention feature re-encoding, multi-branch ROI classification, and online hard example mining in a Faster R-CNN–style pipeline [37], as well as cascaded designs combining Mask R-CNN candidate selection/instance segmentation with stacked CNN ensembles and meta-classification [39]. Transfer learning and feature fusion were recurrent implementation motifs, including COCO-pretrained Faster R-CNN with a ResNeXT-101-32×8d-FPN backbone [40] and a two-stream detector fusing U-Net-derived segmentation features with RGB features inside a Faster R-CNN detection pipeline [84]. Later studies emphasized mitigation of small-object failure modes and multi-step refinement, including a Faster R-CNN variant with dilated convolutions and an optimized RPN [43], and a multi-step two-stage pipeline combining detector variants with post-detection window relocation, center adjustment, classifier re-scoring, and weighted confidence fusion [82]. A further trend was explicit detector → re-classifier coupling, such as Cascade R-CNN–based detection followed by deep re-classification [55], and Mask R-CNN instance segmentation/detection with ROI-based patch extraction and mask-derived re-annotation into COCO-style labels [59].

#### 3.5.2. One-Stage Object Detectors (n = 11)

**Figure 8:**
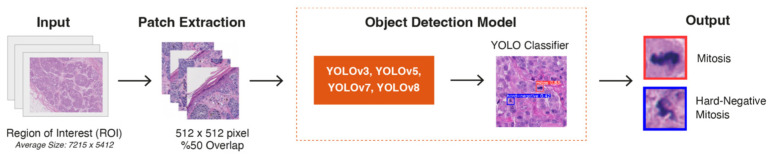
Example of one-stage object detectors (Topuz et al. [45]).

Eleven studies primarily relied on single-pass object detection formulations, dominated by YOLO-family models but also including transformer-based detector variants and coarse-to-refined one-stage frameworks. Within this family, methodological variation was driven by three recurring axes: (i) detector architecture selection and modification, (ii) preprocessing designed to address stain/scanner variability, and (iii) strategies to improve small-object assignment and manage false positives. Representative examples of preprocessing-conditioned one-stage detection included a YOLOv4 pipeline evaluated under two different input variants (raw RGB vs. stain-unmixed inputs via color deconvolution/stain unmixing) [60], and a YOLOv8-based approach augmented with HRNet and HSV plus wavelet preprocessing [56]. Several studies implemented detector–recognition separation even while remaining one-stage in the localization step, such as a scaled-YOLOv4 detection model paired with a separate recognition classifier [71], and a RetinaNet-style coarse detector followed by an explicit classifier to remove hard negatives/false positives, with additional attention/normalization and hybrid anchor design for adaptive scale handling [75]. Comparative and ensemble-centric evaluations were also reported, including tile-level detection studies benchmarking YOLOv5 alongside Faster R-CNN within a common platform [12], and evaluations spanning multiple YOLO variants (YOLOv3/YOLOv4-Scaled/YOLOv5/YOLOR) with an averaging-based ensemble [69] and broader YOLO model comparisons across YOLOv3/YOLOv5/YOLOv7/YOLOv8, including “tiny” or low-parameter variants [45] (Figure 8). Recent entries included specialized transformer-based and DETR-style detectors, such as a transformer-backbone one-stage detector with a dense–sparse hybrid label assignment strategy [61] and a DETR-based detector variant with a CSPResNeXt backbone, reduced decoder layers, and CIoU loss [79]. Finally, later pipelines emphasized modular detector-plus-classifier staging while retaining a one-stage detector as the localization core, such as YOLOv7 for cell detection followed by ConvNeXt for classification [81], and architectural augmentation of a YOLO11-L baseline via modified backbone and neck components, including hypergraph convolution [73].

#### 3.5.3. Dense Segmentation/Heatmap-Based Detection (n = 9)

**Figure 9:**
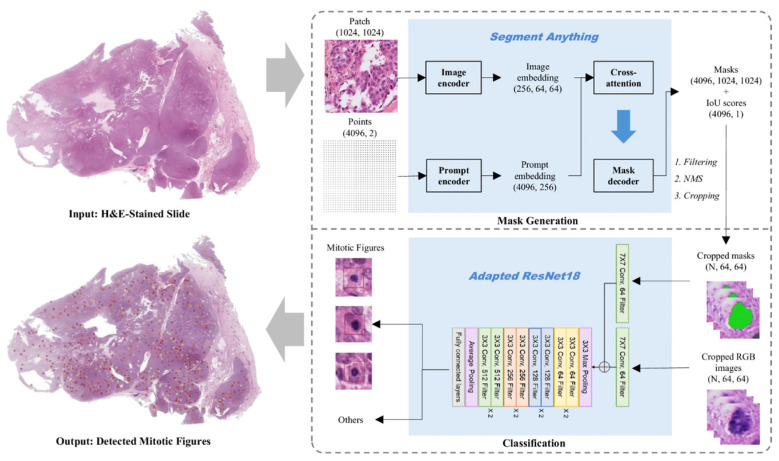
Example of dense segmentation/heatmap-based detection (Shen et al. [80]).

Nine studies framed mitosis detection as dense prediction—semantic segmentation, heatmap regression, or pixel-wise scoring—followed by centroid/peak extraction or subsequent lightweight decision rules. Early pixel-wise FCN-style approaches emphasized multi-scale context aggregation and feature fusion, including a dense FCN detector with two-scale branches, multi-level fusion, and modified multi-scale loss alongside stain normalization and blue-ratio preprocessing [32]. Multi-task and partially supervised designs were also prominent: one end-to-end multi-task pipeline combined R2U-Net segmentation for region-annotated data with regression-style detection for point annotations and an additional classifier stage for final decisions [35], while a partially supervised formulation used parallel FCNs for weak-label and strong-label streams with weight transfer and fused segmentation maps [17]. Subsequent dense prediction studies increasingly focused on the supervision bottleneck—deriving usable pixel-level targets from sparse annotations—such as a weak-to-strong conversion strategy that generated pixel labels from centroid annotations prior to training a ResNet-based FCN segmentation network [16], and a two-phase design in which U-shaped segmentation generated bounding-box “strong labels” from weak point labels before training an R-CNN detector [38]. Encoder–decoder variants evolved toward stronger backbones and segmentation heads, including the replacement of FCN with DeepLabv3+ followed by centroid extraction [18], and hybrid architectures combining CNN and transformer elements within a U-Net-style framework for small-object localization maps [50]. More recent dense-prediction pipelines incorporated additional post-processing or downstream discrimination modules while preserving dense localization as the first stage, including a two-stage design where SAM-based cell segmentation was followed by a ResNet18 classifier [80] (Figure 9), and a candidate proposal FCN coupled to centroid-offset regression and a second-stage classifier using a ResNet50 + RBF layer with spectral-clustering centroids and alternating cluster ↔ train steps [19]. A dense segmentation study explicitly incorporating domain generalization via FFT/Fourier augmentation [53] is discussed under the robustness-focused family below, reflecting its stated methodological emphasis on generalization under domain shift.

#### 3.5.4. Candidate-Based Cascades (Proposal + Classifier/Refinement; n = 5)

**Figure 10:**
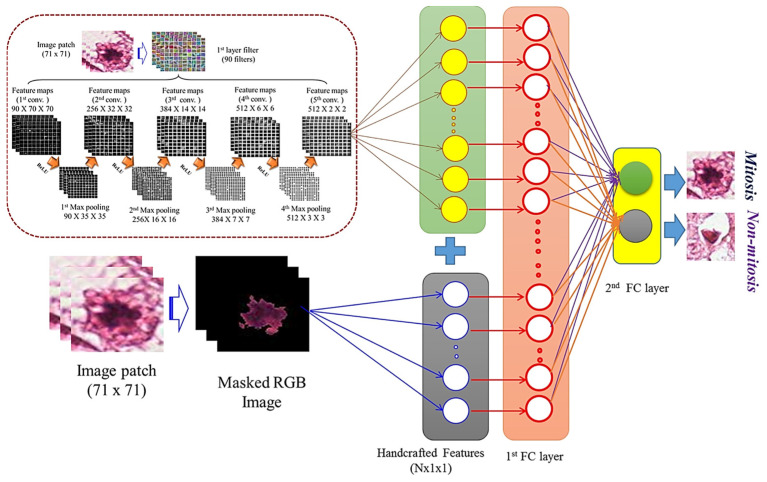
Example of candidate-based cascades (Saha et al. [62]).

Five studies explicitly decoupled candidate proposal generation (high-recall, often heuristic-driven) from a second-stage refinement model tasked with rejecting hard negatives. Candidate generation commonly leveraged color transforms and morphological operators, followed by patch classification with deep or hybrid feature representations. For example, one pipeline generated candidates via blue-ratio transform, morphology, and centroid detection, then performed CNN-based patch classification while merging handcrafted morphological/textural/intensity features into the network [62] (Figure 10). A related approach combined blue-ratio and Otsu-based candidate detection with stain normalization, then classified candidates using a deep CNN applied to Haar wavelet–decomposed patches [33]. Other cascades emphasized discriminative refinement through metric learning, including candidate proposal via morphology/Otsu/connected components followed by a Wide-ResNet feature extractor trained with classification loss plus triplet-based metric learning and hard-negative mining [36]. Hybrid candidate generation and ensemble decision-making were also reported, such as candidate nuclei segmentation using blue-ratio transform with StarDist followed by hybrid handcrafted and CNN deep features and ensemble voting across classical learners [44].

Finally, segmentation-to-classification cascades appeared in which stain normalization/enhancement/augmentation preceded U-Net segmentation trained with BCE + Dice loss, and segmented ROIs were then classified using a modified VGG16 classifier [63]. Across this family, the unifying design rationale was to maximize sensitivity at the proposal stage and shift specificity to a dedicated refinement classifier trained to discriminate morphologically similar mimics.

#### 3.5.5. Handcrafted/Classical ML Pipelines (n = 3)

**Figure 11:**
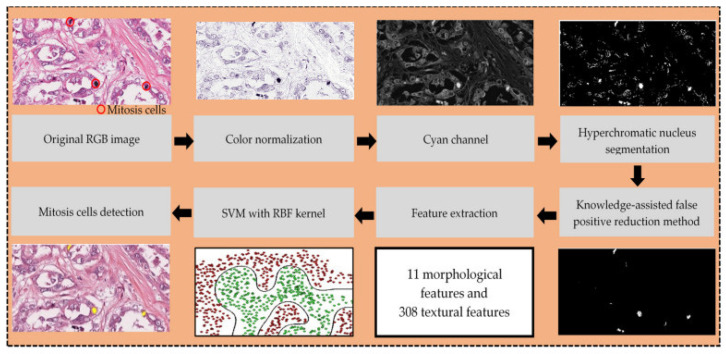
Example of handcrafted/classical ML pipelines (Tan et al. [65]).

Three studies retained predominantly feature-engineered pipelines with conventional machine learning classifiers, typically coupled with rule-based filtering. One pipeline used blue-ratio thresholding with morphological processing and size filtering for candidate segmentation, extracted morphological/texture/color wavelet histogram features, optionally applied SMOTE, and reported SVM as the best-performing classifier [64]. Another emphasized handcrafted texture representations (ELBP/GLCM/LTP, including a “GLCM doughnut” feature) combined within a weighted multi-classifier ensemble (SVM, Random Forest, Naive Bayes) with majority voting [42]. A third used stain normalization followed by K-means-based hyperchromatic nucleus segmentation and domain-knowledge false-positive reduction before classification using handcrafted morphology/texture features and an RBF-kernel SVM [65] (Figure 11). In the context of the 2018–2025 evidence base, these pipelines represent a minority approach relative to deep learning paradigms, but they remain methodologically distinct due to explicit reliance on engineered descriptors and rule-driven decision points.

#### 3.5.6. Deep Feature Extraction/Patch Classification (Incl. Hybrids; n = 12)

**Figure 12:**
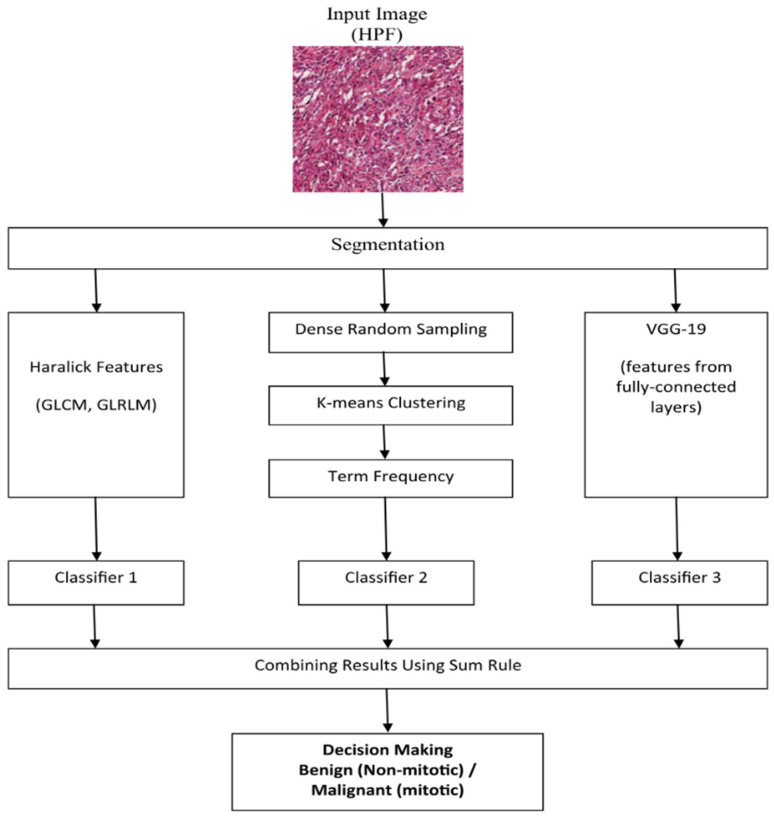
Example of Deep feature extraction/patch classification (Dhivya and Vasuki et al. [41]).

Twelve studies formulated mitosis detection primarily as patch/cell-level classification or as deep feature extraction followed by a classical decision layer, including both CNN- and transformer-based representations and a small number of non-traditional model families. Several pipelines combined preprocessing and candidate localization heuristics with deep feature extraction and downstream classical classifiers, such as stain normalization plus multi-thresholding nuclei localization followed by VGG-based feature extraction, PCA/feature selection, and Random Forest classification [66], and dense sliding-window inference using a rotation-equivariant Group-CNN patch classifier with peak (local-maximum) detection [85]. Pure deep patch classifiers were also reported, including CNN architectures trained with Adam and cross-entropy (ESPNet and DenseNet161) [57], as well as hybrid feature-ensemble pipelines that compared handcrafted texture features, bag-of-features representations, and CNN features (VGG19), each classified with an RBF-kernel SVM [41] (Figure 12). Additional hybridization was observed in pipelines using segmentation-driven candidate extraction (median filtering, k-means segmentation, morphology) followed by ROI patch extraction, feature computation, and an imbalance-aware classifier such as RUSBoost [68], and in pipelines that extracted multi-CNN features (VGG16/ResNet50/DenseNet201), optionally applied PCA, and used linear SVM/LinearSVC as the final classifier [48]. Later studies included patch/image-level binary classification using hybrid Conv–Transformer backbones under color normalization and augmentation (ConvMixer, CoatNet) [58], as well as feature-extraction strategies that explicitly combined multiple pretrained backbones and optimization heuristics [78] and transfer-learning designs incorporating skip connections and hybrid feature selection/optimization [49]. Transformer-centric patch classification was represented by a ViT attention network with quadratic feature aggregation/classification and contrast/luminance-enhanced stain normalization [51]. Notably, one study operationalized mitosis-related prediction via vision–language models, formulating inference as image captioning and VQA using CLIP and BLIP with metadata inputs (e.g., tumor type/species/scanner) [72]. Collectively, this family reflects sustained interest in modular “representation + decision layer” designs, including classical ML heads and emerging foundation-style representations.

#### 3.5.7. Representation Learning/Domain Adaptation–Generalization (n = 7)

**Figure 13:**
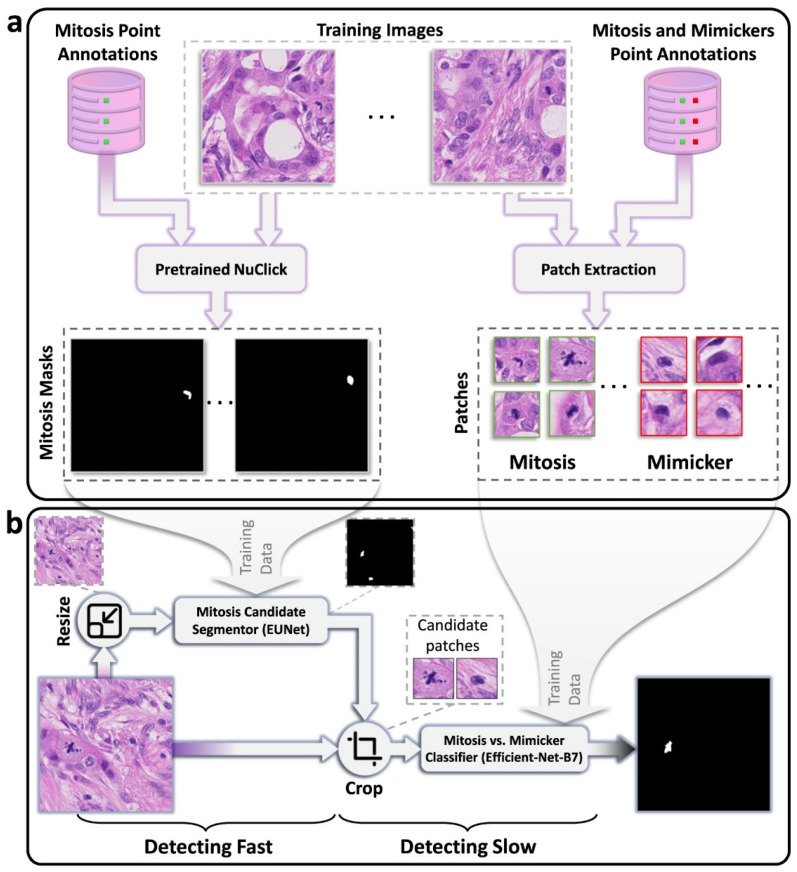
Example of representation learning/domain adaptation–generalization. (**a**) data preprocessing, including generation of mitosis masks and mitosis/mimicker patches; and (**b**) the proposed “Detecting Fast” and “Detecting Slow” pipelines for candidate segmentation and refinement (Jahanifar et al. [47]).

Seven studies foregrounded robustness to domain shift through explicit representation learning or domain adaptation–generalization mechanisms, spanning candidate cascades, dense segmentation, and detector-centric heads. One early example incorporated contrastive similarity learning within a two-stage cascade: candidate generation via multi-scale CNNs followed by a Siamese-style similarity model trained with a contrastive (large-margin) loss to remove hard mimics [31]. Unsupervised domain adaptation was operationalized via adversarial learning with gradient reversal/H-divergence, combining a domain-adaptive Mask R-CNN proposal stage with a domain-adaptive classifier [70]. Contrastive pretraining for downstream histopathology tasks was represented by an unsupervised contrastive learning framework with semantically relevant positives and a hybrid CNN plus multi-scale Swin Transformer backbone, followed by fine-tuning for detection [52]. Domain generalization was also explicitly integrated into dense prediction, including a semantic segmentation pipeline trained with pseudo pixel-level labels and attention mechanisms and augmented for generalization via FFT/Fourier-based transformations [53], as well as a two-stage pipeline combining rapid candidate segmentation with deep refinement under an explicit domain generalization framing [47] (Figure 13). Self-supervised transfer learning was instantiated in a two-stage design in which candidate extraction via traditional segmentation preceded classification using either supervised training or a self-supervised DINO backbone (frozen backbone with a linear classifier) across multiple backbones [46]. Finally, domain alignment regularization was embedded directly within an anchor-free detector, described as a one-stage model with a dynamic depth-wise convolution backbone and GRL-style domain alignment regularizers [54]. Taken together, these studies demonstrate that, within the extracted evidence base, robustness-oriented methodological contributions were implemented across multiple architectural scaffolds rather than being confined to a single detector family.

Table 3 maps each study’s method family to its evaluation regime, showing that custom hold-out dominates across all families. Official/challenge tests are less common but offer the strongest basis for direct between-study comparison when protocols match. External validation and explicit DG are rare and mostly appear in robustness-oriented work and a small subset of detector-centric studies—supporting our protocol-stratified synthesis (official/challenge vs. within-study hold-out/CV evidence).

### 3.6. Public Benchmarks and Multi-Domain Datasets: Outcomes Stratified by Dataset and Evaluation Type

**Figure 14:**
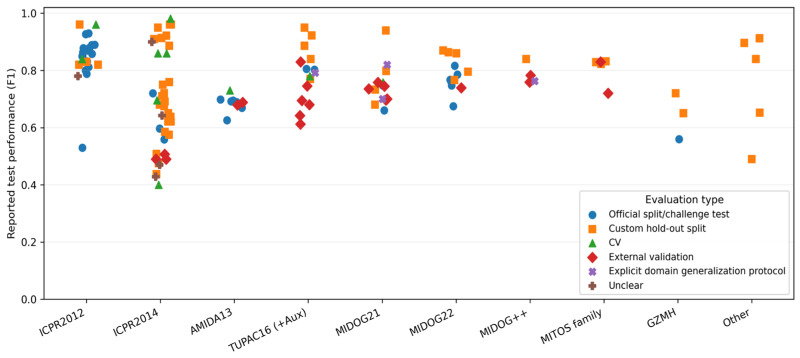
Scatter plot of reported F1 by dataset family, stratified by evaluation type. Each point represents one reported study-level result; differences across evaluation regimes indicate that F1 values are not directly comparable without protocol stratification.

To ensure protocol-consistent interpretation, we synthesized outcomes per dataset and stratified within each dataset by evaluation type, always in the following order: (i) official split/challenge testing, (ii) custom hold-out splits, (iii) cross-validation, (iv) explicit domain generalization (DG) protocols, (v) external validation where the dataset serves as a target, and finally (vi) unclear or insufficiently specified protocols. Because performance estimates are strongly protocol-dependent, values are not compared across evaluation categories, and hold-out/CV outcomes are interpreted primarily as within-study evidence. This is consistent with the separation visible in Figure 14, where reported F1 differs systematically across evaluation regimes within the same dataset families.

Table 4 maps method families to the major public and multi-domain benchmarks used as test sets, indicating where empirical evidence is available for each dataset–method combination. Legacy benchmarks (ICPR2012/ICPR2014 and AMIDA13) account for most evaluations across a broad range of method families, while the MIDOG datasets (MIDOG21/22 and MIDOG++) are used more frequently in one-stage and robustness-oriented (DA–DG/representation learning) studies. This distribution motivates our dataset-by-dataset synthesis and highlights that conclusions about cross-domain robustness are supported by a smaller and more methodologically concentrated evidence base than conclusions drawn from legacy within-domain benchmarks.

**Figure 15:**
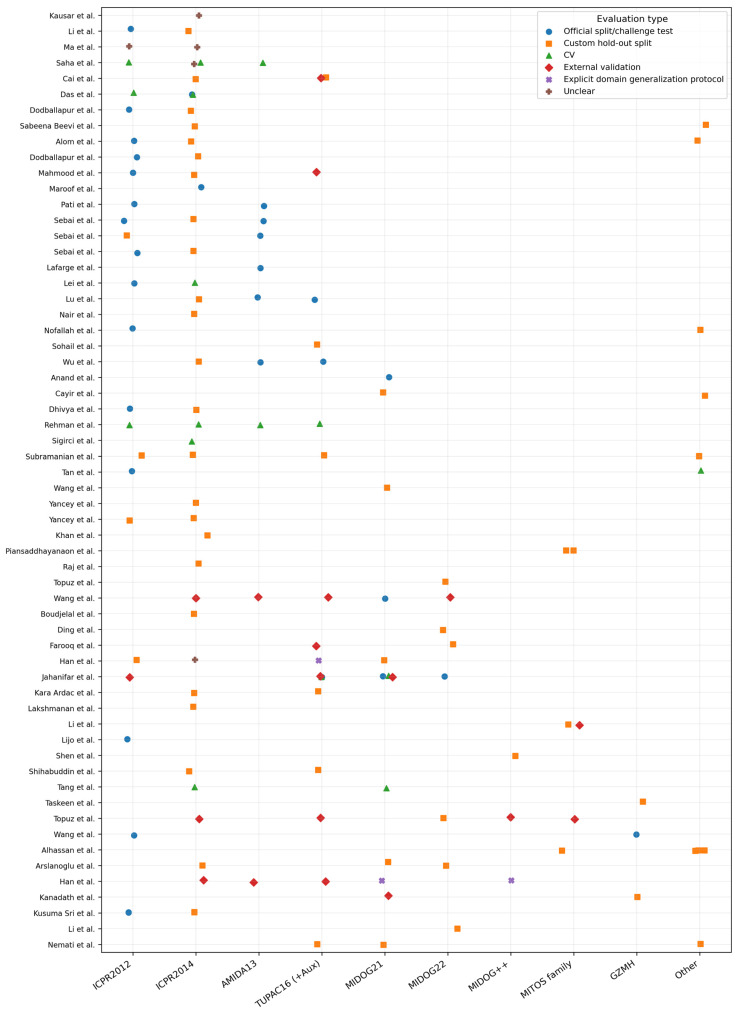
Evidence map of dataset families by evaluation type (study × dataset family matrix). Visual overview of which dataset families were evaluated under which evaluation regimes across included studies. Markers denote evaluation type (e.g., official/challenge, custom hold-out). Multiple markers per study can appear when a study reports results on multiple dataset families and/or under multiple evaluation regimes. Studies are identified on the y-axis as Author and correspond to references [12,13,14,15,16,17,18,19,22,32,33,34,35,36,37,38,39,40,41,42,43,44,45,46,47,48,49,50,51,52,53,54,55,56,57,58,59,60,61,62,63,64,65,66,67,68,69,70,71,72,73,76,78,79,80,81,82,83,84,85].

Figure 15 provides a compact, study-level summary of the entire evidence base: it maps each included study to the dataset families it evaluated and the evaluation regimes used (e.g., official/challenge, custom hold-out), making clear that many papers report multiple test targets and sometimes multiple protocols within the same manuscript.

#### 3.6.1. ICPR2012 (MITOS2012): Outcomes by Evaluation Type

Dataset characteristics: ICPR2012 is a legacy breast mitosis benchmark frequently reported with an official split/challenge-style evaluation in the included literature. In the extraction table it is repeatedly associated with Aperio XT scanning (test dataset field) and is treated as a primary legacy benchmark alongside ICPR2014 (Supplementary Data S2).

Official split/challenge test: Seventeen studies reported ICPR2012 performance under an official split/challenge test regime, with F1 ranging from 0.53 to 0.93, indicating substantial dispersion despite nominally standardized testing. Two-stage detector or detector → verification designs reported mid-to-high values, including DeepMitosis (F1 = 0.832) [15], multi-stage Faster R-CNN refinement (F1 = 0.858) [14], and a mask-driven two-stage hybrid (F1 = 0.87) [34]. Candidate-based cascades and metric-learning refinement reported F1 = 0.812 [36]. Dense/weak-supervision segmentation-style approaches also appear in this official-test stream, including F1 = 0.788 [17] and F1 = 0.878 [35]. At the upper end of this evidence stream, the highest reported official test value reached F1 = 0.93 [51]. The lowest reported value (F1 = 0.53) occurred in an explicit unsupervised domain-adaptive configuration [70], emphasizing that adaptation settings are not directly comparable to standard within-domain test evaluation.

Custom hold-out splits: Three studies reported ICPR2012 outcomes under custom hold-out splits, with F1 spanning 0.82–0.96. These include a tile-level detection pipeline (F1 = 0.82) [12] and a YOLO-family real-time detection/ensemble configuration reaching F1 = 0.96 [69]. Because the studies do not standardize split construction, these results are interpreted as within-study outcomes rather than as benchmark-comparable evidence.

Cross-validation: One classical handcrafted feature study reported ICPR2012 cross-validation performance with F1 = 0.96 [42]. As with other CV evidence, the estimate reflects the study’s fold construction and sampling procedure rather than a shared benchmark protocol.

External validation (target-side reporting)*:* ICPR2012 appears as an external validation target in one multi-dataset robustness-oriented pipeline, which reported F1 = 0.736 and F1 = 0.745 under different external-transfer directions/configurations [47]. These values are interpreted strictly in their external-transfer context and not pooled with within-benchmark official results.

Unclear protocol evidence: One study reports ICPR2012: F1 = 0.78 under an explicitly unclear split designation in the extraction table [31].

#### 3.6.2. ICPR2014 (MITOS-ATYPIA-14): Outcomes by Evaluation Type

Dataset characteristics: ICPR2014 is characterized as a larger/more challenging dataset, and reports a size of 2400/992 (train/test) in the dataset-level splits. The ambiguity note further emphasizes that ICPR2014 test ground truth is reserved, making strict comparability contingent on confirmed organizer-scored evaluation (Supplementary Data S2).

Official split/challenge test: Within the ICPR2014 (MITOS-ATYPIA-14) evidence coded as official split/challenge-style, extracted F1 values ranged from 0.559 to 0.72. However, for several papers, the governance of “test” evaluation could not be confirmed as organizer-scored hidden test performance, and these cases are documented here explicitly. Das et al. evaluated a deep CNN–based mitosis detector with scanner-stratified reporting and obtained F1 = 0.597 on the Hamamatsu test subset and F1 = 0.559 on the Aperio test subset. While the study follows the official split structure, it reports that test annotations/ground truth were generated post-challenge rather than being the original organizer-held hidden labels, and that evaluation was performed by the authors on this test set [33]. Maroof et al. reported F1 = 0.72 using a handcrafted-feature pipeline with oversampling-based imbalance handling (SMOTE-style) and a conventional classifier, but although the manuscript labels the benchmark as “ICPR2014,” the dataset description is more consistent with ICPR2012 than with ICPR2014 [64]. This result is therefore recorded for completeness but flagged as dataset-identity ambiguous and is not treated as definitive ICPR2014 evidence. Across ICPR2014-associated publications more generally, “test” terminology is sometimes used without confirming organizer-governed hidden-test scoring; these instances are retained with explicit ambiguity notes to prevent over-interpretation in cross-study comparison.

Custom hold-out splits: ICPR2014 hold-out evidence is extensive and heterogeneous: 23 studies reported custom hold-out outcomes with F1 ranging from 0.437 to 0.96, reflecting wide sensitivity to split design, candidate definitions, and scanner/domain composition. Reported values include F1 = 0.437 (DeepMitosis) [15], F1 = 0.585 (modified Faster R-CNN light-head) [13], F1 = 0.691 (multistage Faster R-CNN proposal + refinement) [14], F1 = 0.621 (weak → strong conversion segmentation) [16], and F1 = 0.73 vs. 0.65 under differing input variants in a YOLOv4 study [60]. At the upper end, multiple studies reported F1 = 0.96 in hold-out regimes, including a hybrid ensemble pipeline [44] and another high report extracted in the table [78]. Because split construction is study-specific, these high values are treated as evidence of achievable within-study performance rather than definitive benchmark dominance.

Cross-validation: Five studies reported ICPR2014 CV results, spanning F1 = 0.40–0.981, reinforcing that cross-validation is not a single standardized evidence stream. The lowest reported value (F1 = 0.40) occurs in a two-stage detection + screening design [37], while the highest (F1 = 0.981) appears in a candidate-detection + wavelet-decomposed CNN pipeline [33]. Additional CV reports include F1 = 0.86 in a handcrafted texture-feature ensemble [42], F1 = 0.86 in a segmentation-driven candidate extraction + RUSBoost classifier pipeline [68], and F1 = 0.696 in a two-stage dense proposal + classifier framework [19]. These values are interpreted strictly within each study’s fold design.

External validation (target-side reporting)*:* ICPR2014 appears as an external target in three robustness-oriented studies, with F1 = 0.49 under external validation in a dense segmentation + DG-augmentation framework trained elsewhere [53], F1 = 0.49 under external validation within a YOLOv7 → ConvNeXt cascade evaluated across multiple targets [81], and F1 = 0.507 reported as external testing in a DG-focused detector framework [54]. These are interpreted solely as external-transfer evidence.

Unclear protocol evidence: Several ICPR2014-linked outcomes were coded as unclear because the manuscripts do not report enough information to map the results to a specific evaluation regime under the operational definitions used in this review. Saha et al. reported F1 = 0.90 for ICPR2014 in a deep-learning study trained across multiple public datasets (ICPR2014/ICPR2012/AMIDA13), but the extraction notes indicate that the evaluation pathway cannot be determined with confidence (organizer-scored hidden test versus an author-defined testing setup) [62]. Ma et al. reported F1 = 0.47 for ICPR2014 using a two-stage cascade combining multi-scale CNN candidate generation with Siamese similarity learning for refinement/verification; however, the split and evaluation governance were insufficiently specified to classify the result as official or custom hold-out [31]. Kausar et al. reported F1 = 0.428 using an FCN-style multi-scale dense prediction formulation, again without reporting a partitioning/governance description that supports confident regime assignment [32]. Han et al. reported F1 = 0.642 while stating that test ground truth is reserved; because test metrics are nevertheless reported without clarifying whether they were obtained via the challenge server, organizer evaluation, or unofficial labels, the outcome is also coded as unclear [61]. These results are retained as separate evidence setting but are not pooled with clearly defined official or hold-out evaluations and are not used for protocol-commensurate performance comparisons.

#### 3.6.3. AMIDA13: Outcomes by Evaluation Type

Dataset characteristics: AMIDA13 includes 12 train/11 test cases with >1000 mitoses, and is consistently treated as a legacy breast benchmark in the extraction table (Supplementary Data S2).

Official split/challenge test: Six studies reported AMIDA13 official-regime outcomes, clustering in a narrow band with F1 = 0.626–0.698. Reported values include F1 = 0.689 [38], F1 = 0.692 [16], and dense/partial supervision variants near the upper end (F1 = 0.698) [17]. A rotation-equivariant Group-CNN approach reported F1 = 0.626 [85]. Across this dataset, methodological diversity does not translate into wide divergence under official testing, suggesting the protocol strongly constrains comparability.

Cross-validation: One handcrafted-feature ensemble study reported F1 = 0.73 under cross-validation [42].

External validation (target-side reporting): AMIDA13 appears as an external target with F1 = 0.679 [53] and F1 = 0.688 [54], both within robustness-oriented evaluation settings rather than AMIDA13′s official benchmark evaluation.

#### 3.6.4. TUPAC16: Outcomes by Evaluation Type

Dataset characteristics: TUPAC16 is an auxiliary mitosis dataset comprising 73 cases and including overlap with AMIDA13 cases. In the extraction table, TUPAC16 is repeatedly used both as a within-dataset benchmark and as an external validation target in multi-domain designs (Supplementary Data S2).

Official split/challenge test: Three studies reported official-regime outcomes on TUPAC16, with F1 = 0.675–0.805. Weak → strong label conversion pipelines reported F1 = 0.805 [16] and F1 = 0.803 [38], while a multi-domain candidate segmentation + refinement pipeline reported F1 = 0.675 under challenge testing [47].

Custom hold-out splits: Six studies reported TUPAC16 hold-out outcomes with F1 = 0.736–0.95, spanning two-stage region/proposal detectors and one-stage Transformer-based detectors. Extracted values include F1 = 0.736 [13], F1 = 0.84 [12], F1 = 0.886 [66], and a DETR-style one-stage formulation reaching F1 = 0.95 [79]. A YOLOv8+HRNet hybrid detector also reports high hold-out performance (F1 = 0.922) [56]. These values are not pooled with official-test evidence due to split heterogeneity.

Cross-validation: Two studies reported TUPAC16 CV results: F1 = 0.78 [42] and F1 = 0.767 [47].

Explicit DG protocol: One study explicitly evaluated a lab-to-lab DG protocol on TUPAC16 (train on Lab1, test on Lab2/3) and reported F1 = 0.792 [61], providing one of the clearest robustness-oriented within-dataset evaluations in the corpus.

External validation (target-side reporting)*:* Four studies reported TUPAC16 as an external target, with F1 spanning 0.642–0.83: F1 = 0.642 [14], F1 = 0.700 [54], F1 = 0.697 under one external-transfer configuration [47], and F1 = 0.83 in a MIDOG22-trained classification-oriented pipeline [67]. These results are interpreted as cross-domain transfer evidence, not as direct TUPAC16 benchmark estimates.

#### 3.6.5. MIDOG21: Outcomes by Evaluation Type

Dataset characteristics: MIDOG21 is a multi-domain challenge dataset acquired across six scanners and comprising > 2500 mitoses in aggregate reporting. Challenge documentation further specifies a training set of 150 labeled and 50 unlabeled cases (images covering 2 mm^2^ regions), containing 1721 mitotic figures and 2714 mimickers. Evaluation of the associated 80-case MIDOG21 test set is conducted via challenge leaderboard submission, as the test data are not publicly accessible. The dataset’s multi-scanner acquisition introduces explicit domain shift, and published evaluation protocols frequently employ leave-one-domain-out or scanner-stratified validation strategies. In the extraction table, MIDOG21 is therefore consistently treated as multi-domain/multi-scanner evidence under organizer-governed challenge evaluation (Supplementary Data S2).

Official split/challenge test: Three studies reported MIDOG21 official challenge evaluation with F1 = 0.66–0.747. A Faster R-CNN-based pipeline reported F1 = 0.66 [40], while higher official results include F1 = 0.747 in two different pipelines: a segmentation-style DG-augmented approach [53] and a multi-domain candidate segmentation + refinement pipeline [47].

Custom hold-out splits: Five studies reported MIDOG21 hold-out outcomes with F1 = 0.68–0.94. These include a two-stage detection → recognition workflow (F1 = 0.68) [71], representation-learning backbone fine-tuning (F1 = 0.7332) [52], and a YOLOv8+HRNet detector reporting F1 = 0.94 [56].

Cross-validation: Two studies reported MIDOG21 CV outcomes: F1 = 0.758 [19] and F1 = 0.785 [47].

Explicit DG protocol: One study reported scanner-held-out DG evaluation on MIDOG21 (held-out 1/3 scanner) with F1 = 0.820 [54]. This constitutes a distinct and more stringent robustness evidence stream compared with within-scanner hold-out or challenge testing.

External validation (target-side reporting): MIDOG21 appears as an external target in three robustness-oriented studies, including F1 = 0.70 [50], F1 = 0.700 reported under a MIDOG21 → target transfer configuration in a DG-focused detector framework [54], and multiple external transfer outcomes reported in a multi-domain refinement pipeline [47], including F1 = 0.697 and F1 = 0.758 under different external-validation configurations. These are interpreted strictly under their transfer design.

#### 3.6.6. MIDOG22: Outcomes by Evaluation Type

Dataset characteristics: The MIDOG22 training set contains 354 labeled images spanning multiple tumor types (canine lung cancer, human breast cancer, canine lymphoma, human neuroendocrine tumor, and canine cutaneous mast cell tumor), with cross-validation commonly performed across training domains. The associated external MIDOG22 test set comprises 100 images from tumor types not represented in the training set, thereby explicitly increasing cross-domain difficulty. As with MIDOG21, acquisition across different scanners introduces domain shift. In the extraction table, MIDOG22 is consistently treated as a multi-domain dataset and frequently appears in cross-dataset or robustness-oriented evaluation designs (Supplementary Data S2).

Official split/challenge test: One study reported MIDOG22 challenge evaluation with F1 = 0.764 [47].

Custom hold-out splits: Six studies reported MIDOG22 hold-out outcomes with F1 = 0.766–0.87. Reported values include F1 = 0.766 in a YOLO variant comparison [45], F1 = 0.795 in a YOLOv7 → ConvNeXt cascade [81], F1 = 0.863 in a modified YOLO11-L formulation [73], and F1 = 0.87 in a classification-oriented pipeline [67]. A candidate-extraction + classifier pipeline reports both supervised and SSL (DINO) variants in the same hold-out regime, with F1 = 0.8390 (supervised) and F1 = 0.8275 (DINO) [46]. These are treated as within-study comparisons.

Cross-validation: One study reported F1 = 0.816 under CV on MIDOG22 [47].

External validation (target-side reporting): One external evaluation reports F1 = 0.7389 on MIDOG22 as an external target [53].

#### 3.6.7. MIDOG++: Outcomes by Evaluation Type

Dataset characteristics: MIDOG++ contains 503 annotated images across seven cancer types and is described as the largest currently available published dataset of mitotic figures. As an extension of the MIDOG multi-domain framework, MIDOG++ preserves scanner and tumor-type heterogeneity and is explicitly designed to support domain generalization research. In the extraction table, MIDOG++ is treated as a multi-domain dataset and is used primarily in robustness and cross-domain evaluation protocols (Supplementary Data S2).

Custom hold-out splits: One study reported a SAM-assisted segmentation → classification pipeline under hold-out evaluation with F1 = 0.84 [80].

External validation: Topuz et al. evaluated a two-stage deep learning architecture on tumor types not encountered during training. When tested on MIDOG++ melanoma images, the model achieved F1 = 0.783, and on MIDOG++ soft tissue sarcoma images, F1 = 0.759 [81]. As these experiments involved testing on a distinct dataset extension containing tumor types and, in part, acquisition centers not included in the MIDOG22 training data, they are coded as external validation evidence rather than custom hold-out evaluation.

Explicit DG protocol: One study reported an explicit domain-held-out evaluation on MIDOG++ (held-out 1/7 domain) with F1 = 0.763 [54]. This DG evidence is interpreted separately from standard hold-out evaluation.

### 3.7. Additional Datasets and Cohorts: Outcomes Stratified by Evaluation Type

#### 3.7.1. MITOS_CMC (CODAEL) and MITOS_CCMCT (ODAEL): Outcomes by Evaluation Type

Dataset characteristics: MITOS_CMC (canine mammary carcinoma; 21 WSIs) and MITOS_CCMCT (canine mast cell tumor; 32 WSIs) are described as WSI datasets designed for mitosis detection. In the extraction table, these datasets are used primarily in two-stage detector → classifier designs.

MITOS_CMC (custom hold-out)**:** Two studies reported MITOS_CMC hold-out outcomes: F1 = 0.823 for a multi-step detector + relocation + re-scoring pipeline [82] and F1 = 0.829 in a Cascade R-CNN + classifier re-scoring design [55].

MITOS_CCMCT (custom hold-out and external): One study reported hold-out performance on MITOS_CCMCT (F1 = 0.832) [82]. Two studies reported MITOS_CCMCT as an external validation: F1 = 0.83 [55] and F1 = 0.72 in a YOLOv7 → ConvNeXt cascade evaluated as an external melanoma target [81].

#### 3.7.2. GZMH: Outcomes by Evaluation Type

Dataset characteristics: The GZMH dataset (Ganzhou Municipal Hospital, China) is used for breast cancer mitosis analysis.

Official split/challenge test: One cascaded coarse-to-refine detector framework reports F1 = 0.56 under official split/challenge-style reporting [75].

Custom hold-out splits: Two studies reported hold-out outcomes on GZMH, spanning F1 = 0.65–0.72, including a Mask R-CNN instance segmentation/detection workflow (F1 = 0.65) [59] and a hybrid CNN–Transformer encoder–decoder dense model (F1 = 0.72) [50].

#### 3.7.3. KMIT, MiDeSeC, MITNET, RCC, CWRU, Melanoma Biopsy WSIs, and Custom Dataset

For these datasets, evidence is sparse (typically single-study reporting) and should be interpreted as study-specific rather than benchmark-comparable.

KMIT appears once under hold-out evaluation with F1 = 0.84 [12]. MiDeSeC appears once under hold-out evaluation with F1 = 0.912 [56]. MITNET appears once with F1 = 0.49 under hold-out evaluation [71]. RCC appears once with F1 = 0.896 under hold-out evaluation in a transfer-learning feature extraction + RF pipeline [66]. CWRU appears once with F1 = 0.652 under hold-out evaluation in a multi-task detection/classification framework [35]. A melanoma biopsy WSI cohort appears once under hold-out validation with very high patch-level classification performance (F1 = 0.968 and F1 = 0.976 for two CNN variants) [57]. Finally, one study reports a Custom dataset under cross-validation with F1 = 0.89 [65].

#### 3.7.4. Accuracy-Only Multi-Dataset Reporting

Alhassan et al. report multi-dataset results under a custom hold-out regime using accuracy (rather than detection F1): 98.8% (MITOS_CCMCT), 98.5% (Mitosis-AIC), 98.3% (Mitosis Detection), and 98.1% (Mitosis and Non-Mitosis) (dataset-wise values reported in their Table 3) [49]. Because these outcomes are not protocol-aligned detection F1 estimates, they are not pooled with the F1-based benchmark synthesis. Although the paper also reports F-measure/F1 (paper’s Table 2), the corresponding dataset and split are not explicitly linked; therefore, we do not attribute an F1 value to a specific dataset in extraction (the numerical proximity suggests it may correspond to MITOS_CCMCT, but this mapping is not stated).

### 3.8. Evaluation Metrics

Across the included studies, F1-score (or F-measure) was the dominant primary metric for mitosis detection, reflecting the strong class imbalance and the need to balance false positives and false negatives. Commonly reported metrics were:

F1-score (F-measure): Harmonic mean of precision and recall, providing a single balanced summary that remains informative under imbalance and aligns with detection-oriented evaluation practice [86,87].

Accuracy: Proportion of correct predictions. It is easy to interpret, but can be misleading under strong class imbalance, and is therefore most informative when reported alongside error-type metrics [86,87].

Sensitivity (Recall): Fraction of true mitoses correctly detected. This metric captures missed mitoses (false negatives) and is critical when under-detection is clinically undesirable [86,87].

Specificity: Fraction of non-mitoses correctly rejected. This reflects control of false positives, which otherwise inflate workload through unnecessary candidate review [86,87].

Precision (Positive Predictive Value): Fraction of predicted mitoses that are correct. Precision quantifies the reliability of positive detections and is particularly informative in candidate-heavy pipelines [86,87].

## 4. Discussion

### 4.1. Principal Findings and Overall Interpretation

This PRISMA-guided systematic review synthesized 66 included publications (2018–2025), comprising 60 method papers on automated mitotic figure detection in H&E histopathology and six dataset/challenge descriptor papers retrieved directly by the same search queries and retained to contextualize benchmark construction and evaluation governance. Across the 60 method studies, three overarching patterns emerge.

First, the field expanded rapidly over the review window, peaking in 2024 (15/60 studies), while maintaining sustained output through 2025 (7/60). Despite this growth, the evidence base remains concentrated in breast histopathology (46/60 studies), with multi-domain designs accounting for a smaller but non-trivial fraction (12/60). Second, the methodological landscape is diverse but structured: most studies target object-level localization/detection (53/60), and the dominant paradigms map to modern detection families (two-stage R-CNN: 13/60; one-stage detectors: 11/60) and dense prediction/heatmap formulations (9/60), complemented by candidate cascades (5/60), patch/feature-based classification pipelines (12/60), classical handcrafted pipelines (3/60), and representation learning/DA–DG–oriented work (7/60). Third—and most consequential for interpretability—reported performance is strongly conditioned on an evaluation regime. At the study level, custom hold-out evaluation was most common (42/60), while official split/challenge testing appeared in 24/60, cross-validation in 8/60, external validation in 9/60, and explicit domain generalization (DG) protocols in only 2/60; 4/60 were coded as unclear due to insufficiently specified evaluation governance.

Together, these findings support a key interpretive conclusion: the literature provides abundant evidence that high performance is achievable under within-study evaluation designs, but the strength of evidence for transfer under domain shift is comparatively sparse and unevenly distributed across datasets and method families. This imbalance matters because mitosis detection is particularly sensitive to variation in scanner characteristics, staining protocols, tissue context, and mimicker prevalence—exactly the factors targeted by external validation and domain-held-out DG designs.

### 4.2. What Methodological Diversity Actually Represents in This Corpus

Across the included studies, methodological “families” are better understood as dominant localization mechanisms, rather than mutually exclusive end-to-end systems. Hybridization is the norm: detector → classifier cascades, segmentation → classification pipelines, and candidate generation, followed by metric learning or verification, appear repeatedly. For that reason, this review’s primary family assignment is intentionally anchored to the mechanism that produces candidate mitosis locations (e.g., proposals for R-CNN–family methods; single-pass detection heads for YOLO/DETR-style one-stage methods; dense maps for segmentation/heatmap methods), rather than downstream refinement components. This distinction is not merely taxonomic—it aligns the discussion with what most directly determines error modes (missed small objects vs. false positives from mimickers) and influences portability across domains.

Within this structure, the data suggest that “new architecture” is not the only driver of apparent progress. Many high-performing systems combine fairly standard backbones with carefully chosen preprocessing, supervision conversion (weak-to-strong), candidate filtering, or multi-stage verification. Conversely, more recent representational shifts, e.g., self-supervised encoders, explicit domain alignment losses, and domain-generalization augmentations, tend to appear most often in the subset of studies that actually test robustness beyond a single dataset. In other words, the methodological frontier in this corpus is increasingly defined by how evidence is generated (evaluation regime and target domains), not only by the model class.

### 4.3. Evaluation Regime as a First-Order Determinant of Comparability

A central contribution of this review is the explicit separation of evaluation regimes, because these regimes correspond to different strengths of evidence. The Results show that even when the same dataset name is used, the reported metric can reflect fundamentally different evaluation governance and data partitioning. This is not a minor reporting detail: protocol heterogeneity can plausibly dominate observed performance differences, especially in benchmarks where case-level leakage, scanner overlap, or site overlap can occur under author-defined splits.

The ICPR2014 evidence stream makes this issue particularly visible. We noted that several papers describe their evaluation as “testing on ICPR2014” and sometimes even restate how the original ICPR2014 test subset was defined, without making clear that the organizer-held hidden test labels were not publicly available to them. This creates the impression of evaluation on the official ICPR2014 test split, although the reported experiments may in fact have been conducted on the public training portion, or on a custom subset derived from it. The consequence is straightforward: such reports cannot automatically be treated as equivalent to organizer-scored challenge outcomes, and any upper-bound values based on ambiguously governed “test” evaluation should be interpreted cautiously in cross-study comparisons. By retaining these results transparently while keeping them protocol-qualified (and, when necessary, separate as unclear), the synthesis avoids overstating what the evidence supports.

More broadly, the distribution of regimes in Table 1 indicates that most of the literature still prioritizes within-dataset estimation (hold-out or CV) over external transfer or domain-held-out testing. This is a critical interpretive constraint: strong within-dataset results demonstrate feasibility and optimization within a dataset’s characteristics, but they do not, by themselves, establish robustness under deployment-relevant shift.

### 4.4. What the Robustness-Oriented Evidence Supports—And What It Does Not

Robustness-oriented evaluation remains a minority practice in this corpus (external validation: 9/60; explicit DG: 2/60), but it is also the most informative for readers concerned with real-world transfer. Importantly, “robustness” is not instantiated consistently across papers. Some studies operationalize it as cross-dataset transfer (training on one named benchmark and testing on another), others as scanner- or lab-held-out protocols (explicit DG), and others as domain adaptation objectives that use unlabeled target data during training. These designs answer different questions, and pooling them would blur interpretation; however, they collectively show that performance estimates tend to be more stringent when evaluation is designed to break within-domain overlap.

A single illustrative example captures the distinction cleanly: Topuz et al. trained on MIDOG2022 and then tested on MIDOG++ extension sets containing tumor types not encountered during training, including melanoma and soft tissue sarcoma images (with part of the sarcoma data coming from a center not included in training) [81]. In our coding, this is external validation evidence because it evaluates transfer to a dataset extension with novel tumor types and, partially, novel acquisition centers. This kind of design supports a stronger claim than a random hold-out split within one dataset family because it actively instantiates the shift that challenges mitosis detectors in practice.

**Figure 16:**
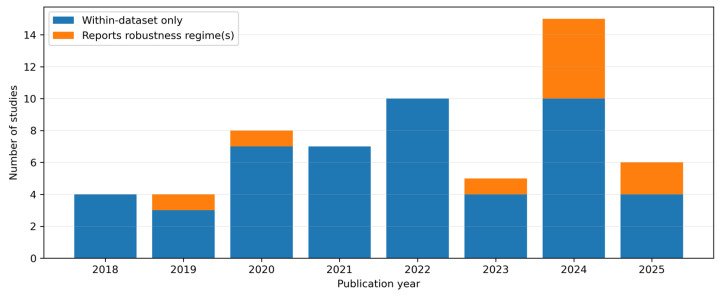
Adoption of robustness-type evaluation over time (study-level): yearly counts of studies reporting only within-dataset testing versus at least one robustness regime (external validation and/or explicit DG).

The explicit DG evidence is even rarer but methodologically clearer: Han et al. report a lab-to-lab protocol on TUPAC16 (training on one lab and testing on others) [61], and Han et al. report scanner/domain-held-out protocols in the MIDOG family (e.g., held-out scanner/domain by design) [54]. These protocols most directly reflect the deployment question: can a system trained on one acquisition domain maintain performance on a genuinely unseen domain without target labels used for fitting? This temporal pattern is summarized in Figure 16, which shows that robustness-type evaluation remains uncommon across 2018–2025, despite increasing in later years.

Finally, domain adaptation evidence should be treated as a distinct category rather than folded into DG or standard evaluation. For example, Dodballapur et al. use adversarial UDA objectives (GRL/H-divergence style) with unlabeled target data [70]. This setting tests a different capability—learning domain-invariant representations via access to target-domain samples during training—and its performance should not be interpreted as directly comparable to a pure domain-generalization setting where target labels and target samples are not used for fitting (or used only in strictly defined ways).

### 4.5. Implications for Readers: How to Interpret Reported Performance in Practice

Because 59/60 algorithmic studies report F1 (and only one study is effectively “accuracy-only” for dataset-aligned reporting), the literature appears superficially metric-aligned. However, metric alignment does not solve protocol alignment. A practical way for readers to use the evidence base is to treat reported results as answers to different questions:

Within-dataset custom hold-out and cross-validation results primarily answer, “How well can this pipeline be optimized on this dataset under this study’s split design?” These results are useful for method development and ablation logic, but they should be interpreted as study-specific unless the split is organizer-defined and comparable across studies.

Official split/challenge test results most directly support between-paper comparisons within a benchmark—but only when organizer governance is clear. Where test governance is ambiguous (as seen for some ICPR2014 reports), conclusions should be tied to the protocol detail, not only to the dataset name.

External validation and explicit DG results answer the most deployment-relevant question: “What happens under shift?” The synthesis shows that this evidence stream exists but is still thin relative to the volume of within-dataset reporting, and it concentrates in multi-domain datasets (MIDOG family) and robustness-oriented method families (representation learning/DA–DG).

This regime-aware framing also clarifies why “best reported F1” should not be treated as a global leaderboard across the field: the apparent upper bound is often a function of evaluation design and dataset composition, not only architecture.

### 4.6. Methodological and Reporting Recommendations Supported by the Extracted Evidence

The evidence base itself motivates several concrete recommendations that would materially improve interpretability and comparability:

First, studies should explicitly enumerate, for each reported result: the named dataset, the exact partitioning regime, and whether evaluation was organizer-governed (challenge server/hidden labels) or author-evaluated. The ICPR2014 ambiguity notes reported in this review show that this distinction is not always recoverable from manuscripts, which directly limits evidence synthesis.

Second, because 70% of studies report ≥2 test datasets and 58.3% report ≥2 training datasets, multi-dataset reporting is already common; what is missing is consistent regime labeling per dataset. A minimal reporting standard would require a dataset-by-dataset mapping of train/test identity and evaluation type aligned with the categories used here.

Third, readers and developers would benefit from routine pairing of within-dataset reporting with at least one robustness-oriented evaluation whenever multi-domain datasets are available. The MIDOG-family resources and veterinary WSI datasets demonstrate that such evaluations are feasible; the limiting factor appears to be reporting practice rather than dataset availability.

Finally, where studies report alternative metrics (e.g., accuracy in classification-only formulations), the dataset and split associated with each metric should be stated explicitly. Our handling of Alhassan et al.’s work [49] illustrates why: without a clear dataset–split mapping for F1, the result cannot be integrated into benchmark-level synthesis even if the paper reports an F-measure table.

### 4.7. Tissue Context, Sampling, and Clinical-Pathology Interpretation

The included literature was dominated by benchmark-driven mitosis detection rather than by tumor-subtype-specific biological interpretation. Although several tissue and domain contexts were represented, the extracted evidence did not support robust conclusions about associations between mitotic characteristics and specific histological subtypes, treatment response, or tumor biology. Most studies reported detection performance at the level of datasets, image patches, ROIs, HPFs, or WSIs, rather than subtype-stratified mitotic morphology or therapy-response endpoints. Therefore, tissue-type observations in this review should be interpreted primarily as evidence of dataset/domain diversity and domain shift, not as evidence of tumor-specific biological correlations.

Image sampling also varied across the included datasets. Several benchmark datasets used HPF- or ROI-level images, including selected regions enriched for mitosis evaluation, whereas fewer datasets supported WSI-scale assessment. This distinction is important because performance on cropped or selected high-mitotic-activity regions may not fully represent end-to-end deployment across entire tumor sections, where tumor detection, hotspot selection, tissue exclusion, and false-positive control must also be solved. Future studies should therefore report whether evaluation was performed on selected mitotic regions, predefined HPFs/ROIs, or entire WSIs, and should distinguish local detection accuracy from full-slide mitotic-counting performance.

### 4.8. Limitations of This Review

This synthesis is constrained by reporting heterogeneity in the primary literature. Even under a standardized extraction template, some studies do not specify evaluation governance or partition provenance sufficiently to classify the regime confidently, leading to the small “unclear” stratum (4/60) that is retained but not pooled. In addition, heterogeneity in datasets, regime definitions, and reporting granularity precluded quantitative meta-analysis; accordingly, this review follows a structured qualitative approach and reports outcomes exactly as stated within protocol-aligned settings. Finally, the scope is restricted to peer-reviewed English-language studies from 2018 to 2025 and to three databases, which may omit relevant non-indexed or non-English contributions.

### 4.9. Future Directions

The extracted evidence suggests that the field’s limiting factor is increasingly not the availability of detector architectures but the generation of comparable, shift-aware evidence. Three directions follow directly from the synthesis. First, robustness-oriented evaluation should become routine: within-dataset estimates should be paired, where possible, with external validation, multi-scanner or multi-laboratory testing, WSI-level evaluation, and domain-held-out testing. Second, protocol reporting should be standardized and explicit, especially for benchmarks with hidden test labels or challenge governance; thresholds and operating points should be fixed before final testing rather than optimized on the test set. Third, emerging representation-centric approaches, including self-supervised encoders, domain-alignment regularization, and promptable segmentation front-ends, should be evaluated primarily on what they promise—transfer under shift—rather than only on peak within-dataset performance.

For clinical translation, future studies should also report outcomes in clinically meaningful terms, including false-positive and false-negative behavior, reproducibility of mitotic counts, threshold stability, and the reliability of grading support across deployment sites. These additions would make reported performance more interpretable for real-world digital pathology workflows and would better distinguish methodological progress from favorable evaluation design.

In tissue contexts with dense lymphocytic infiltrates or abundant karyorrhectic/apoptotic debris, future algorithms should be evaluated with explicit hard-negative annotations and error stratification. False positives should be reported not only as aggregate counts but, where possible, by morphologic category, such as lymphocytes, apoptotic/karyorrhectic bodies, inflammatory cells, or staining/crush artifacts. False negatives should be interpreted in relation to missed mitotic figures within the clinically relevant counting region. Such reporting would be particularly important for lymphoma-like or highly cellular tumors, where the visual background may differ substantially from the breast-cancer benchmarks that dominate the current evidence base.

## 5. Conclusions

This PRISMA-guided systematic review synthesized evidence from 60 method papers (2018–2025) on automated mitotic figure detection in H&E histopathology and six dataset/challenge descriptor papers retrieved by the same search strategy and retained to contextualize benchmark construction and evaluation governance. Across the extracted evidence table, three evidence-grounded conclusions emerge.

First, the literature demonstrates that strong performance is achievable, but reported values are not inherently comparable across papers unless interpreted jointly with a dataset identity and evaluation regime. Although F1 was the dominant reported outcome (59/60 studies; one study reported accuracy-only in a way that could not be protocol-aligned for F1 pooling), the review identified substantial evaluation heterogeneity: custom hold-out testing was most common (42/60), followed by official split/challenge-style reporting (24/60), cross-validation (8/60), external validation (9/60), and explicit domain generalization (DG) protocols (2/60). In 4/60 studies, evaluation was coded as unclear/insufficiently specified, reflecting cases where partition provenance or organizer-governed scoring could not be verified. This protocol diversity means that “best” values across papers often represent different strengths of evidence (within-study optimization vs. organizer-governed testing vs. cross-domain transfer), rather than directly comparable estimates of model capability.

Second, the evidence base remains benchmark-concentrated and breast-dominant, shaping what can be concluded with confidence. Most evaluations rely on a small set of recurrent public benchmarks—particularly ICPR2014 (37/60) and ICPR2012 (25/60), followed by TUPAC16 (16/60) and AMIDA13 (10/60)—while multi-domain datasets from the MIDOG family (MIDOG21: 11/60; MIDOG22: 8/60; MIDOG++: 3/60) increasingly anchor robustness-oriented studies. Importantly, for ICPR2014, the review documented recurring ambiguity around “test” evaluation governance in some reports (e.g., lack of confirmation of organizer-scored hidden-test evaluation or use of post-challenge labels). This reinforces the necessity of protocol-qualified interpretation, particularly when interpreting upper-bound values.

Third, the methodological landscape reflects recurring design patterns rather than a single dominant solution, and robustness-oriented evidence remains comparatively limited. Across the mutually exclusive taxonomy, the most frequent paradigms were two-stage R-CNN–family detectors (13/60), one-stage detectors (11/60), deep feature/patch-classification hybrids (12/60), and dense segmentation/heatmap approaches (9/60), with smaller shares for candidate cascades (5/60), classical handcrafted pipelines (3/60), and representation learning/DA–DG–focused studies (7/60). Hybrid systems (e.g., detector → classifier, segmentation → classifier) were common, indicating that practical gains often derive from pipeline composition and supervision strategy as much as from backbone choice. However, the subset of studies that directly test cross-domain behavior (external validation or explicit DG) is still small relative to the volume of within-dataset reporting, limiting how strongly the literature can currently support claims about deployment-level generalization.

Overall, the reviewed evidence supports that automated mitosis detection can reach high performance under specific experimental conditions, but reliable translation requires stronger, more standardized evidence of generalization. Based on the extracted corpus, the most impactful improvements for future work are: (i) routine pairing of within-dataset results with external validation and/or domain-held-out DG when feasible, (ii) explicit, dataset-by-dataset reporting of train/test identity and evaluation governance (official vs. author-defined; organizer-scored vs. author-evaluated), and (iii) harmonized outcome reporting that avoids ambiguous “test” language and enables protocol-consistent synthesis across benchmarks.

## Figures and Tables

**Table 1:** Evidence table. Baseline characteristics of included algorithmic studies (N = 60).

Characteristic	n/N (%)	Representative Studies
Total included algorithmic studies	60	
Publication year		
2018	4/60 (6.7%)	Li et al. [15]; Ma et al. [31]; Kausar et al. [32]
2019	4/60 (6.7%)	Das et al. [33]; Dodballapur et al. [34]; Cai et al. [13]
2020	8/60 (13.3%)	Alom et al. [35]; Pati et al. [36]; Mahmood et al. [14]
2021	7/60 (11.7%)	Lei et al. [37]; Lu et al. [38]; Sohail et al. [39]
2022	10/60 (16.7%)	Anand et al. [40]; Dhivya and Vasuki et al. [41]; Rehman et al. [42]
2023	5/60 (8.3%)	Khan et al. [43]; Raj et al. [44]; Topuz et al. [45]
2024	15/60 (25.0%)	Arslanoglu et al. [46]; Jahanifar et al. [47]; Shihabuddin et al. [48]
2025	7/60 (11.7%)	Alhassan et al. [49]; Kanadath et al. [50]; Kusuma Sri et al. [51]
Domain/tissue (as reported)		
Breast	46/60 (76.7%)	Li et al. [15]; Das et al. [33]; Rehman et al. [42]
Multi-domain	12/60 (20.0%)	Wang et al. [52]; Wang et al. [53]; Han et al. [54]
Breast; Canine mammary carcinoma	1/60 (1.7%)	Sohail et al. [39]
Mast cell tumor (canine); skin	1/60 (1.7%)	Li et al. [55]
Task type		
Object-level detection	53/60 (88.3%)	Li et al. [15]; Lei et al. [37]; Nemati et al. [56]
Patch/cell-level classification	7/60 (11.7%)	Nofallah et al. [57]; Boudjelal et al. [58]; Kusuma Sri et al. [51]
Primary method family (mutually exclusive taxonomy)		
Two-stage region/proposal detectors (R-CNN family)	13/60 (21.7%)	Li et al. [15]; Sohail et al. [39]; Taskeen et al. [59]
One-stage object detectors	11/60 (18.3%)	Nair et al. [60]; Han et al. [61]; Nemati et al. [56]
Dense segmentation/heatmap-based detection	9/60 (15.0%)	Kausar et al. [32]; Wu et al. [16]; Kanadath et al. [50]
Candidate-based cascades (proposal + classifier/refinement)	5/60 (8.3%)	Saha et al. [62]; Pati et al. [36]; Lijo et al. [63]
Handcrafted/classical ML pipelines	3/60 (5.0%)	Maroof et a. [64]; Rehman et al. [42]; Tan et al. [65]
Deep feature extraction/patch classification (incl. hybrids)	12/60 (20.0%)	Sabeena Beevi et al. [66]; Farooq et al. [67]; Kusuma Sri et al. [51]
Representation learning/ domain adaptation-generalization	7/60 (11.7%)	Ma et al. [31]; Das et al. [33]; Han et al. [54]
Evaluation types reported (not mutually exclusive at study level)		
Official split/challenge test reported	24/60 (40%)	Li et al. [15]; Nofallah et al. [57]; Kusuma Sri et al. [51]
Custom hold-out split reported	42/60 (70.0%)	Li et al. [15]; Khan et al. [43]; Nemati et al. [56]
Cross-validation reported	8/60 (13.3%)	Saha et al. [62]; Sigirci et al. [68]; Tang et al. [19]
External validation reported	9/60 (15.0%)	Cai et al. [13]; Jahanifar et al. [47]; Kanadath et al. [50]
Explicit DG/domain-held-out evaluation reported	2/60 (3.3%)	Han et al. [61]; Han et al. [54]
Unclear/insufficiently specified evaluation reported	4/60 (6.7%)	Ma et al. [31]; Saha et al. [62]; Han et al. [61]
Test datasets used (not mutually exclusive)		
ICPR2014	37/60 (61.7%)	Li et al. [15]; Yancey et al. [69]; Kusuma Sri et al. [51]
ICPR2012	25/60 (41.7%)	Li et al. [15]; Dodballapur et al. [70]; Kusuma Sri et al. [51]
TUPAC16	16/60 (26.7%)	Cai et al. [13]; Farooq et al. [67]; Nemati et al. [56]
MIDOG21	11/60 (18.3%)	Cayir et al. [71]; Jahanifar et al. [47]; Nemati et al. [56]
AMIDA13	10/60 (16.7%)	Saha et al. [62]; Wu et al. [16]; Han et al. [54]
MIDOG22	8/60 (13.3%)	Topuz et al. [45]; Ding et al. [72]; Li et al. [73]
Other test datasets beyond the six above	16/60 (26.7%)	e.g., MITOS_CCMCT; GZMH; MIDOG++; MiDeSeC; KMIT; RCC; melanoma biopsy WSIs;
Scope of dataset usage (derived from canonical dataset parsing)		
≥2 test datasets reported per study	42/60 (70.0%)	
≥2 train datasets reported per study	35/60 (58.3%)	
Primary test outcome metric (as recorded)		
F1 reported	59/60 (98.3%)	
Accuracy-only (no F1)	1/60 (1.7%)	Alhassan et al. [49]

**Table 2:** Upper-bound F1 by dataset and evaluation type (best within each setting). Cell format: best F1 (Author–Year; method family); ties shown as A/B. Family abbreviations: 2S-RCNN = Two-stage R-CNN; 1S-DET = One-stage detector; DENSE = Dense segmentation/heatmap; CASCADE = Candidate cascade; HAND = Handcrafted/classical ML; PATCH = Deep feature/patch classification; REPR = Representation learning/DA–DG.

Dataset	Official/Challenge	Custom Hold-Out	Cross-Validation	Explicit DG	External Validation	Unclear
ICPR2012 [5]	0.930 [51]; PATCH	0.960 [69]; 1S-DET	0.960 [42]; HAND	—	0.745 [47]; REPR	0.780 [31]; CASCADE
ICPR2014 [20]	0.72 * [64]; HAND	0.960 [78]; PATCH/0.960 [44]; CASCADE	0.981 [33]; CASCADE	—	0.507 [54]; REPR	0.900 [62]; CASCADE
AMIDA13 [10]	0.698 [17]; DENSE	—	0.730 [42]; HAND	—	0.688 [54]; REPR	—
TUPAC16 [74]	0.805 [16]; DENSE	0.950 [79]; 1S-DET	0.780 [42]; HAND	0.792 [61]; 1S-DET	0.830 [67]; PATCH	—
MIDOG21 [21]	0.747 [47]; REPR/ 0.747 [53]; DENSE	0.940 [56]; 1S-DET	0.785 [47]; REPR	0.820 [54]; REPR	0.758 [47]; REPR	—
MIDOG22 [22]	0.764 [47]; REPR	0.870 [67]; PATCH	0.816 [47]; REPR	—	0.739 [53]; DENSE	—
MIDOG++ [26]	—	0.840 [80]; DENSE	—	0.763 [54]; REPR	melanoma: 0.783 sarcoma: 0.759 [81]; 1S-DET	—
MITOS_CMC [76]	—	0.829 [55]; 2S-RCNN	—	—	—	—
MITOS_CCMCT [77]	—	0.832 [82]; 2S-RCNN	—	—	0.830 [55]; 2S-RCNN	—
GZMH [75]	0.560 [75]; 1S-DET	0.720 [50]; DENSE	—	—	—	—

* Paper reports on ‘ICPR2014′-dataset, but the described dataset matches ICPR2012, not ICPR2014. The results for this setting are discussed separately.

**Table 3:** Evidence map linking method families to evaluation regimes (study-level coverage; N = 60). Cells are n (row %), where row % is within the method family. Evaluation regimes are not mutually exclusive at the study level (a study can report multiple regimes).

Method Family (Mutually Exclusive Primary Taxonomy)	Official Split/Challenge Test	Custom Hold-Out	Cross-Validation	External Validation	Explicit DG Protocol	Unclear Protocol
Two-stage proposal detectors (R-CNN) (n = 13)	6 (46.2%)	11 (84.6%)	1 (7.7%)	3 (23.1%)	0 (0.0%)	0 (0.0%)
One-stage detectors (n = 11)	1 (9.1%)	10 (90.9%)	0 (0.0%)	1 (9.1%)	1 (9.1%)	1 (9.1%)
Dense segmentation/ heatmap (n = 9)	5 (55.5%)	7 (77.8%)	1 (11.1%)	1 (11.1%)	0 (0.0%)	1 (11.1%)
Candidate cascades (proposal → classifier/ refinement) (n = 5)	3 (60.0%)	1 (20.0%)	2 (40.0%)	0 (0.0%)	0 (0.0%)	1 (20.0%)
Handcrafted/classical ML (n = 3)	2 (66.7%)	0 (0.0%)	2 (66.7%)	0 (0.0%)	0 (0.0%)	0 (0.0%)
Deep feature/patch classification (incl. hybrids) (n = 12)	4 (33.3%)	10 (83.3%)	1 (8.3%)	1 (8.3%)	0 (0.0%)	0 (0.0%)
Representation learning/DA–DG (n = 7)	3 (42.9%)	3 (42.9%)	1 (14.3%)	3 (42.9%)	1 (14.3%)	1 (14.3%)

**Table 4:** Evidence map linking method families to key public benchmarks and multi-domain datasets (study-level coverage; N = 60). Cells are n (row %) within method family. Datasets are not mutually exclusive at the study level (a study can test multiple datasets).

Method Family	ICPR2014	ICPR2012	AMIDA13	TUPAC16	MIDOG21	MIDOG22	MIDOG++
Two-stage proposal detectors (R-CNN) (n = 13)	8 (61.5%)	5 (38.5%)	0 (0.0%)	3 (23.1%)	1 (7.7%)	0 (0.0%)	0 (0.0%)
One-stage detectors (n = 11)	6 (54.5%)	4 (36.4%)	0 (0.0%)	5 (45.5%)	3 (27.3%)	3 (27.3%)	1 (9.1%)
Dense segmentation/ heatmap (n = 9)	6 (66.7%)	3 (33.3%)	4 (44.4%)	2 (22.2%)	2 (22.2%)	0 (0.0%)	1 (11.1%)
Candidate cascades (n = 5)	3 (60.0%)	4 (80.0%)	2 (40.0%)	0 (0.0%)	0 (0.0%)	0 (0.0%)	0 (0.0%)
Handcrafted/ classical ML (n = 3)	2 (66.7%)	3 (100.0%)	1 (33.3%)	1 (33.3%)	0 (0.0%)	0 (0.0%)	0 (0.0%)
Deep feature/patch classification (n = 12)	7 (58.3%)	3 (25.0%)	1 (8.3%)	2 (16.7%)	0 (0.0%)	2 (16.7%)	0 (0.0%)
Representation learning/ DA–DG (n = 7)	5 (71.4%)	3 (42.9%)	2 (28.6%)	3 (42.9%)	5 (71.4%)	3 (42.9%)	1 (14.3%)

## Data Availability

No new data were created or analyzed in this study.

## Supplementary Materials

The following supporting information can be downloaded at: https://www.mdpi.com/article/10.3390/biomedicines14061369/s1, Supplementary Data S1: Extraction Table; Supplementary Data S2: Extraction Table for the public datasets and challenge benchmarks used for training and evaluation of mitosis detection in this review (2018–2025); Supplementary Data S3: Search Queries; Supplementary Data S4: Quality assessment methods; Supplementary Data S5: Supplementary Methods S1. Evaluation-regime classification rules; Supplementary Methods S2: Method-family decision rules.

## Abbreviations

The following abbreviations are used in this manuscript:

AI	Artificial Intelligence
AMIDA	Assessment of Mitosis Detection Algorithms
AP	Average Precision
AUC	Area Under the Curve
AUROC	Area Under the Receiver Operating Characteristic Curve
CNN	Convolutional Neural Network
CV	Cross-Validation
DETR	Detection Transformer
DG	Domain Generalization
DL	Deep Learning
FCN	Fully Convolutional Network(s)
F1	F1-score
GT	Ground Truth
H&E	Hematoxylin and Eosin
HPF	High-Power Field(s)
ICPR	International Conference on Pattern Recognition
mAP	Mean Average Precision
MIDOG	Mitosis Domain Generalization Challenge
ML	Machine Learning
OSF	Open Science Framework
PRISMA	Preferred Reporting Items for Systematic Reviews and Meta-Analyses
QUADAS-2	Quality Assessment of Diagnostic Accuracy Studies 2
R-CNN	Region-based Convolutional Neural Network
ROI	Region of Interest
RPN	Region Proposal Network
SVM	Support Vector Machine
SWiM	Synthesis Without Meta-analysis
TUPAC	Tumor Proliferation Assessment Challenge
UDA	Unsupervised Domain Adaptation
WSI	Whole-Slide Image(s)
YOLO	You Only Look Once
