# Supplementary material for: Recent Advances in Automated Mitosis Detection in Digital Pathology: A PRISMA-Guided Systematic Review with Evaluation-Regime Stratification (2018–2025)
Source: Biomedicines. 2026 Jun 17;14(6):1369. doi: 10.3390/biomedicines14061369 (PMC13296622; doi:10.3390/biomedicines14061369)
Supplement: Supplementary file 1 [file biomedicines-14-01369-s001.zip › biomedicines-4334488-supplementary/Supplementary Files/Supplementary Data S1.pdf]

Supplementary Data A: Extraction Table

Study	Domain/ tissue	Train datasets	Test datasets	Evaluation type	Method category	Task type	Best reported result (test set)
Li (2018)	Breast	ICPR2012, ICPR2014	ICPR2012 (Aperio XT scanner), ICPR2014	ICPR2012: Official split/challenge test, ICPR2014: Custom hold-out split	DeepMitosis: Multi-stage deep learning pipeline: detection (Faster R-CNN/RPN) → segmentation (FCN for box estimation from centroids) → verification (ResNet).	Object-level detection	ICPR2012 (DeepDet): F1 = 0.832; ICPR2014 (DeepDet+Seg+Ver): F1 = 0.437
Ma (2018)	Breast	ICPR2012, ICPR2014: (Aperio Scanscope XT)	ICPR2012, ICPR2014 (Aperio Scanscope XT)	ICPR2012: Unclear, ICPR2014: Unclear[1]	MSSN: Two-stage cascade: (1) FNR-model candidate generation using multi-scale CNNs; (2) FPR-model similarity learning (Siamese-style network) with contrastive (large-margin) loss to remove false positives (hard mimics).	Object-level detection	ICPR2012: F1 = 0.78; ICPR2014: F1 = 0.47
Kausar (2018)	Breast	ICPR2014 (Apero-XT scanner)	ICPR2014 (Apero-XT scanner)	ICPR2014: Unclear[2]	MFF-CNN: Pixel-wise FCN-style detector (multi-scale fused FCN): (AlexNet/CaffeNet-style FCN) with two-scale branches + multi-level feature fusion+ modified multi-scale loss + multi-step fine-tuning; stain normalization + blue-ratio preprocessing; FC layers replaced by 1×1 conv for dense prediction; upsampling to full resolution.	Object-level detection	F1 = 0.428
Saha (2018)	Breast	ICPR2014; ICPR2012; AMIDA13	ICPR2014; ICPR2012; AMIDA13	ICPR2014: CV, ICPR2012: CV, AMIDA13: CV; ICPR2014[3]: Unclear	Two-stage detector → classifier: Candidate generation (blue-ratio transform, morphology, centroid detection) + patch classification (CNN with 5 conv layers + pooling + ReLU + 2 FC layers, plus handcrafted (morphological/textural/intensity) features merged into the network)	Object-level detection	CV: F1 = 0.90; ICPR2014 (Unclear split): F1 = 0.90
Das (2019)	Breast	ICPR2012, ICPR2014	ICPR2012; ICPR2014: Hamamatsu & Aperio;	ICPR2012: CV, ICPR2014: Official split/challenge test[4]; ICPR2014: CV	Candidate detection (blue-ratio + Otsu) + stain normalization + deep CNN on 2-level Haar wavelet decomposed patches (81×81 → 21×21; best with 3×3 kernels).	Object-level detection	ICPR2012: F1 = 0.84; ICPR2014 (test, Hamamatsu): F1 = 0.597; ICPR2014 (test, Aperio): F1 = 0.559; ICPR2014 (5-fold CV): F1 = 0.981
Dodballapur (2019)	Breast	ICPR2012 (train Mask R-CNN for mask generation); ICPR2014 (train classifier)	ICPR2012, ICPR2014	ICPR2012: Official split/challenge test, ICPR2014: Custom hold-out split	Two-stage, mask-driven hybrid: Stage 1 Mask R-CNN (ResNet-50 backbone) for high-recall candidate masks + boxes; Stage 2 classifier combining hand-crafted mask-based features + deep features from Xception (ImageNet pre-trained; ablation with VGG-16) → FC layers for mitotic vs non-mitotic classification.	Object-level detection	ICPR2012 (Xception): F1 = 0.87; ICPR2014 (VGG-16): F1 = 0.68
Cai (2019)	Breast	TUPAC16, ICPR2014	TUPAC16; ICPR2014	TUPAC16: Custom hold-out split; external validation, ICPR2014: Custom hold-out split	Two-stage region-based object detector: modified Faster R-CNN (light head) + ResNet-101 backbone.	Object-level detection	TUPAC16: F1 = 0.736; ICPR2014: F1 = 0.585; External (best): F1 = 0.612 (ICPR2014´s Hamamatsu → TUPAC16)
Sabeena Beevi (2019)	Breast	ICPR2014 “in-group” folders A03/A04/A07/A10; Clinical RCC dataset (India)	ICPR2014, RCC	ICPR2014: Custom hold-out split, RCC: Custom hold-out split	Two-stage: stain normalization + KHA multi-thresholding nuclei localization → VGGNet transfer learning feature extractor → PCA → feature selection → Random Forest classifier.	Object-level detection	ICPR2014: F1 = 0.886; RCC: F1 = 0.896
Alom (2020)	Breast	ICPR2012, ICPR2014, CWRU (Case Western Reserve University)	ICPR2012, ICPR2014, CWRU (Case Western Reserve University)	ICPR2012: Official split/challenge test, ICPR2014: Custom hold-out split, CWRU: Custom hold-out split	MitosisNet: End-to-end multi-task pipeline: (1) R2U-Net segmentation for region-annotated data; (2) R2U-Net regression (UD-Net) for point-annotated detection; (3) IRRCNN classifier; global/local confidence analysis for final decision.	Object-level detection	ICPR2012: F1 = 0.878; ICPR2014: F1 = 0.759; CWRU: F1 = 0.652
Pati (2020)	Breast	ICPR2012, AMIDA13	ICPR2012, AMIDA13	ICPR2012: Official split/challenge test, AMIDA13: Official split/challenge test	Candidate-based detection + deep metric-learning refinement: color normalization + blue-ratio preprocessing → morphological+Otsu+connected-components to propose nuclei candidates → Wide-ResNet feature extractor trained with classification loss + metric learning (triplet) + hard-negative mining / sampling to classify mitosis vs non-mitosis at candidate locations.	Object-level detection	(Using all training data, joint loss + DWS): ICPR2012: F1 = 0.812; AMIDA13: F1 = 0.669
Mahmood (2020)	Breast	ICPR2012, ICPR2014	ICPR2012, ICPR2014, TUPAC16	ICPR2012: Official split/challenge test, ICPR2014: Custom hold-out split, TUPAC16: External validation	Multistage deep learning: Faster R-CNN candidate detection + deep CNN refinement/classification.	Object-level detection	ICPR2012: F1 = 0.858; ICPR2014: F1 = 0.691; TUPAC16: F1 = 0.642
Dodballapur (2020)	Breast	Two training setups (unsupervised target): (1) Source: ICPR2012 Scanner A (Aperio XT) with masks + labels; Target: ICPR2014 train split (Aperio XT) unlabeled (domain loss only). (2) Source: ICPR2012 Scanner A labeled; Target: ICPR2012 Scanner H (Hamamatsu) unlabeled (domain loss only).	ICPR2014, ICPR2012	ICPR2014: Custom hold-out split, ICPR2012: Official split/challenge test	Dual-stage unsupervised domain adaptation (adversarial GRL / H-divergence): Stage 1 domain-adaptive Mask R-CNN (ResNet-50) for mask + bbox proposals; Stage 2 domain-adaptive VGG19 classifier.	Object-level detection	ICPR2012 (domain adaptive): F1 = 0.53; ICPR2014 (domain adaptive): F1 = 0.71

1 Challenge dataset referenced, but the paper does not explicitly state the train/test partition used for the reported results; results are reported only as “on the ICPR2012/ICPR2014 dataset

2 Official split reported, but evaluation described as preliminary on public data; organizer-scored hidden test not confirmed.

3 They reference MITOS-ATYPIA-14 (ICPR2014) evaluation criteria and give train/test frame counts, but do not specify whether results were produced via the official hidden test protocol/server.

4 The study used the official MITOS-ATYPIA-14 (ICPR2014) split, but the test annotations/ground truth were generated post-challenge (i.e., not the original challenge test labels). Performance was independently evaluated by the authors on that test set.

Study	Domain/ tissue	Train datasets	Test datasets	Evaluation type	Method category	Task type	Best reported result (test set)
Maroof (2020)	Breast	ICPR2014	ICPR2014	ICPR2014: Official split/challenge test [5]	Hybrid handcrafted + ML pipeline: candidate segmentation (blue-ratio thresholding + morphology + size filtering) → handcrafted features (morphological + texture + color wavelet histogram features) → SMOTE (optional) → classifier (SVM best).	Object-level detection	SVM + SMOTE: F1 = 0.72
Sebai (2020b) (MaskMitosis)	Breast	ICPR2012, ICPR2014	ICPR2012, ICPR2014	ICPR2012: Official split/challenge test, ICPR2014: Custom hold-out split	MaskMitosis: two-stage Mask R-CNN for detection + instance segmentation.	Object-level detection	ICPR2012: F1 = 0.863; ICPR2014: F1 = 0.475
Sebai (2020) (PartMitos)	Breast	ICPR2012, ICPR2014, AMIDA13	ICPR2012, ICPR2014, AMIDA13	ICPR2012: Official split/challenge test, ICPR2014: Custom hold-out split, AMIDA13: Official split/challenge test	PartMitos: partially supervised deep learning with two parallel FCNs (weak-label stream + strong-label stream) + weight transfer + fusion of segmentation maps.	Object-level detection	ICPR2014: F1 = 0.575; AMIDA13: F1 = 0.698; ICPR2012: F1 = 0.788
Sebai (2020a)	Breast	AMIDA13, ICPR2012	AMIDA13, ICPR2012	AMIDA13: Official split/challenge test, ICPR2012: Custom hold-out split	Improved SegMitos: semantic segmentation (DeepLabv3+ replacing FCN) → centroid extraction.	Object-level detection	ICPR2012: F1 = 0.820; AMIDA13: F1 = 0.695
Lafarge (2021)	Breast	AMIDA13	AMIDA13	AMIDA13: Official split/challenge test	Rotation-equivariant Group-CNN (SE(2,N) G-CNN) patch classifier; dense sliding-window inference + peak (local-max) detection.	Object-level detection	F1 = 0.626
Lei (2021)	Breast	ICPR2012	ICPR2012; ICPR2014	ICPR2012: Official split/challenge test, ICPR2014: CV	Two-stage detection + screening: VGG16 backbone + RPN (Faster R-CNN-style proposals) + spatial attention module (feature re-encoding) + multi-branch classification subnet (ROI Pooling branch + position-sensitive ROI Pooling branch) + OHEM; centroid-based evaluation.	Object-level detection	ICPR2012: F1 = 0.85; ICPR2014: F1= 0.400
Wu (2021)	Breast	AMIDA13, ICPR2014, TUPAC16	AMIDA13, ICPR2014, TUPAC16	AMIDA13: Official split/challenge test, ICPR2014: Custom hold-out split, TUPAC16: Official split/challenge test	Weak→strong label conversion + segmentation (FCN): pixel-level label generation from centroids using gradient-changing threshold (HSV V-channel, Otsu, threshold decay with distance); then ResNet-based FCN (ResNet-50/101 with last two downsamples removed + extra conv layers + upsampling).	Object-level detection	AMIDA13 (ResNet-50): F1 = 0.692; ICPR2014 (ResNet-101): F1 = 0.621; TUPAC16 (ResNet-50): F1 = 0.805
Lu (2021)	Breast	AMIDA2013, ICPR2014, TUPAC16	AMIDA2013, ICPR2014, TUPAC16	AMIDA13: Official split/challenge test, ICPR2014: Custom hold-out split, TUPAC16: Official split/challenge test	Two-phase pipeline: U-shaped segmentation generates bbox "strong labels" from weak point labels → R-CNN detector.	Object-level detection	AMIDA2013: F1 = 0.689; ICPR2014: F1 = 0.621; TUPAC16: F1 = 0.803
Nair (2021)	Breast	ICPR2014	ICPR2014	ICPR2014: Custom hold-out split	One-stage object detection (YOLOv4) with two input variants: raw RGB vs stain-unmixed (color deconvolution / stain unmixing).	Object-level detection	RGB model: F1 = 0.73; stain-unmixed model: F1 = 0.65
Nofallah (2021)	Breast	Melanoma skin biopsy WSIs (6 cases)	Melanoma skin biopsy WSIs; ICPR2012	Melanoma skin biopsy WSIs: Custom hold-out split, ICPR2012: Official split/challenge test	Patch-level CNN classification trained with Adam + cross-entropy; models: ESPNet and DenseNet161.	Patch/cell-level classification	Melanoma val: (ESPNet): F1 = 0.968, (DenseNet161): F1 = 0.976; ICPR2012: (DenseNet161): F1 = 0.927, (ESPNet): F1 = 0.890
Sohail (2021)	Breast	TUPAC16, ICPR2014, ICPR2012	TUPAC16	TUPAC16: Custom hold-out split	Two-stage cascade (proposal → classification): Macenko stain normalization + patching; Mask R-CNN for candidate mitosis selection / instance segmentation (hard-negative undersampling); heterogeneous stacked ensemble (5 custom CNN base models → MLP meta-classifier) for mitosis vs non-mitosis classification.	Object-level detection	F1 = 0.77
Cayir (2022)	Breast	MIDOG21, ICPR2014, MITNET	MIDOG21, MITNET	MIDOG21: Custom hold-out split, MITNET: Custom hold-out split	Two-stage pipeline: MITNET-det (scaled-YOLOv4 with CSPDarknet + PANet for nucleus detection) + MITNET-rec classifier (mitosis vs non-mitosis).	Object-level detection	MIDOG21: F1 = 0.68; MITNET: F1 = 0.49;
Dhivya (2022)	Breast	ICPR2014, ICPR2012	ICPR2014, ICPR2012	ICPR2014: Custom hold-out split, ICPR2012: Official split/challenge test	Feature-ensemble approach: handcrafted texture features + Bag-of-Features (BoF) + CNN features (VGG19 with fine-tuned FC layers); each feature set classified using nonlinear SVM (RBF).	Object-level detection	ICPR2012 (HC+BoF+CNN): F1 = 0.85; ICPR2014 (HC+BoF+CNN): F1 = 0.72
Anand (2022)	Breast	MIDOG21	MIDOG21	MIDOG21: Official split/challenge test	Object detection: Faster R-CNN with transfer learning from COCO; ResNeXT-101-32×8d-FPN backbone.	Object-level detection	F1 = 0.66
Rehman (2022)	Breast	ICPR2012, ICPR2014, AMIDA13, TUPAC16	ICPR2012, ICPR2014, AMIDA13, TUPAC16	ICPR2012: CV, ICPR2014: CV, AMIDA13: CV, TUPAC16: CV	Handcrafted texture features (ELBP/GLCM/LTP incl. "GLCM doughnut") + weighted multi-classifier ensemble (SVM, Random Forest, Naive Bayes) + majority voting.	Patch/cell-level classification	ICPR2012: F1 = 0.96; ICPR2014: F1 = 0.86; AMIDA13: F1 = 0.73; TUPAC16: F1 = 0.78
Sigirci (2022)	Breast	ICPR2014	ICPR2014	ICPR2014: CV	Segmentation-driven candidate extraction + patch classification: median filtering → k-means segmentation + morphological cleanup → window/ROI patch extraction around cellular structures → handcrafted or CNN feature	Object-level detection	Deep (CNN features + RUSBoost): F1 = 0.86

[5] Paper reports on 'ICPR2014'-dataset, but the described dataset matches ICPR2012, not ICPR2014.

Study	Domain/ tissue	Train datasets	Test datasets	Evaluation type	Method category	Task type	Best reported result (test set)
					extraction → RUSBoost classifier (imbalance handling).		
Subramanian (2022)	Breast	ICPR2012, KMIT (75 WSIs)	ICPR2012, ICPR2014, TUPAC16, KMIT	ICPR2012: Custom hold-out split, ICPR2014: Custom hold-out split, TUPAC16: Custom hold-out split, KMIT: Custom hold-out split	Tile-level object detection: Faster R-CNN and YOLOv5; web-based platform.	Object-level detection	ICPR2012 (Faster R-CNN): F1 = 0.82; ICPR2014 (Faster R-CNN): F1 = 0.75; TUPAC16 (Faster R-CNN): F1 = 0.84; KMIT (YOLOv5): F1 = 0.84
Tan (2022)	Breast	ICPR2012; Custom dataset (Malaysia)	ICPR2012; Custom dataset	ICPR2012: Official split/challenge test, Custom dataset: CV	Knowledge-/rule-assisted classical pipeline: stain normalization → K-means-based hyperchromatic nucleus segmentation → domain-knowledge false-positive reduction → handcrafted morphology+texture features → SVM (RBF) classifier.	Object-level detection	ICPR2012: F1 = 0.889; Custom dataset: F1 = 0.89
Wang (2022)	Multi-domain	SSL pretraining: unlabeled patches; detection fine-tuning: MIDOG21	MIDOG21 (mitosis detection)	MIDOG21: Custom hold-out split	CTransPath: unsupervised contrastive learning with semantically-relevant positives (SRCL) + hybrid CNN + multi-scale Swin Transformer backbone; downstream via fine-tuning for detection.	Object-level detection	MIDOG21 (Faster R-CNN with CTransPath encoder): F1 = 0.7332
Yancey (2022a)	Breast	ICPR2014	ICPR2014	ICPR2014: Custom hold-out split	Two-stream / feature-fusion detection: Faster R-CNN detector + U-Net segmentation feature extractor; fusion of U-Net segmentation features with RGB image features within the detection pipeline.	Object-level detection	F1 = 0.508
Yancey (2022b)	Breast	ICPR2014, ICPR2012	ICPR2014, ICPR2012	ICPR2014: Custom hold-out split, ICPR2012: Custom hold-out split	Real-time object detection with YOLO family (YOLOv3, YOLOv4-Scaled, YOLOv5 p5, YOLOR) + parallel ensemble (YOLOv5m-p5 + YOLOR averaged predictions).	Object-level detection	ICPR2012 (YOLOv5-p5 + YOLOR): F1 = 0.96; ICPR2014 (YOLOR): F1 = 0.95
Khan (2023)	Breast	ICPR2014	ICPR2014	ICPR2014: Custom hold-out split	SMDetector: Faster R-CNN + dilated convolutions in backbone; optimized RPN for small objects.	Object-level detection	F1 = 0.638
Piansaddhayanaon (2023)	Mast cell tumor (canine); skin	MITOS_CMC (CODAEL), MITOS_CCMCT (ODAEL)	MITOS_CMC (CODAEL), MITOS_CCMCT (ODAEL)	MITOS_CMC: Custom hold-out split, MITOS_CCMCT: Custom hold-out split	ReCasNet: Two-stage pipeline: (1) detector (RetinaNet / Faster R-CNN / Cascade R-CNN / YOLOF backbones tried) → (2) window relocation (border FP re-inference) → (3) object center adjustment (regression + aux class head) → (4) classifier re-scoring + active-learning data selection (detector–classifier disagreement); weighted confidence fusion.	Object-level detection	MITOS_CCMCT (EfficientNet-B4 classifier + Faster R-CNN detector): F1 = 0.832; MITOS_CMC (EfficientNet-B4 classifier + Faster R-CNN detector): F1 = 0.823
Topuz (2023)	Multi-domain	MIDOG22	MIDOG22	MIDOG22: Custom hold-out split	Single-stage object detection (YOLO family): YOLOv3, YOLOv5, YOLOv7, YOLOv8 (COCO-pretrained variants; best-performing vs tiny/low-parameter variants).	Object-level detection	Best performer (YOLOv8x): F1 = 0.766
Raj (2023)	Breast	ICPR2014	ICPR2014	ICPR2014: Custom hold-out split	Candidate nuclei segmentation via blue-ratio transform + StarDist; hybrid features (handcrafted + CNN deep features) + ensemble ML voting (RF + SVM + XGBoost).	Object-level detection	F1 = 0.96
Wang (2023)	Multi-domain	MIDOG21	MIDOG21, AMIDA13, ICPR2014, TUPAC16-auxiliary, MIDOG22	MIDOG21: Official split/challenge test, AMIDA13: External validation, ICPR2014: External validation, TUPAC16-Auxiliary: External validation, MIDOG22: External validation	FMDet semantic segmentation pipeline: pseudo pixel-level labels + U-Net-like encoder–decoder with attention (SE-ResNeXt50 encoder + SK-attention decoder), trained with Focal + Dice loss; domain generalization via FFT/Fourier augmentation.	Object-level detection	MIDOG21: F1 = 0.747; AMIDA13: F1 = 0.679; ICPR2014: F1 = 0.49; TUPAC16-aux (Centers 2&3): F1 = 0.7458; TUPAC16-aux (Centers 1–3): F1 = 0.6946; MIDOG22: F1 = 0.7389
Boudjelal (2024)	Breast	ICPR2014	ICPR2014	ICPR2014: Custom hold-out split	Two-stage pipeline: (1) training with color normalization + augmentation; (2) "mitosis detection" as patch/image-level binary classification using hybrid Conv+Transformer backbones (ConvMixer, CoatNet).	Patch/cell-level classification	ICPR2014 (ConvMixer): F1 = 0.650; ICPR2014 (CoatNet): F1 = 0.675
Farooq (2024)	Multi-domain	MIDOG22	MIDOG22, TUPAC16	MIDOG22: Custom hold-out split, TUPAC16: External validation	Deep learning classification pipeline (inspired by EfficientNet, ResNet, and dilated convolutions; first layers adapted from EfficientNet-B0) with generalization & interpretability focus.	Patch/tile-level classification	MIDOG22: F1 = 0.87; TUPAC16: F1 = 0.83
Han (2024)	Breast	TUPAC16	TUPAC16, ICPR2012, ICPR2014, MIDOG21	TUPAC16: Explicit domain generalization protocol (train on Lab1, test on Lab2/3), ICPR2012: Custom hold-out split, MIDOG21: Custom hold-out split, ICPR2014: [6] Unclear	SFHLA: One-stage object detection with Transformer backbone + Single-Level Feature (SLF) + Dense–Sparse Hybrid Label Assignment (HLA).	Object-level detection	TUPAC16: F1 = 0.792; ICPR2012: F1 = 0.830; ICPR2014: F1 = 0.642 ; MIDOG21: F1 = 0.798
Jahanifar (2024)	Multi-domain	MIDOG22, MIDOG21	MIDOG22, MIDOG21, TUPAC16, ICPR2012	MIDOG22: Official split/challenge test, MIDOG21: Official split/challenge test; External validation; CV, TUPAC16:	MDFS: Two-stag → EUNet (fast candidate segmentation) + EfficientNet-B7 (candidate refinement) + domain generalization.	Object-level detection	Challenge test: MIDOG21: F1=0.747, MIDOG22: F1=0.764, TUPA16: F1=0.675; External validation: TUPAC16→MIDOG21: F1=0.758, MIDOG21→TUPAC16: F1=0.697,

[6] Authors state test GT is reserved; nevertheless report test metrics; unclear whether obtained via challenge server, organizer-provided evaluation, or unofficial labels.

Study	Domain/ tissue	Train datasets	Test datasets	Evaluation type	Method category	Task type	Best reported result (test set)
				Official split/challenge test; External validation; CV, ICPR2012: External validation			MIDOG21→ICPR2012: F1=0.736, TUPAC16→ICPR2012: F1=0.745; CV: MIDOG21: F1= 0.785, TUPCA16: F1 = 0.767, MIDOG22: F1 = 0.816
Kara Ardac (2024)	Breast	ICPR2014, TUPAC16	ICPR2014, TUPAC16	ICPR2014: Custom hold-out split, TUPAC16: Custom hold-out split	Mi-DETR: DETR-based detector: CSPResNeXt backbone + reduced decoder layers + CIoU loss.	Object-level detection	ICPR2014: F1 = 0.921; TUPAC16: F1 = 0.950
Lakshmanan (2024)	Breast	ICPR2014	ICPR2014	ICPR2014: Custom hold-out split	Improved DeepMitosisNet: Deep learning model-based feature extraction: pre-trained CNN architecture (Resnet50, InceptionV3, Faster-RCNN, Deep CNN) optimized with Teaching–Learning-Based Optimizer (TLBO).	Object-level detection	F1 = 0.96
Li (2024)	Breast; Canine mammary carcinoma	MITOS_CMC (CODAEL)	MITOS_CMC (CODAEL), MITOS_CCMCT (ODAEL)	MITOS_CMC: Custom hold-out split, MITOS_CCMCT: External validation	DilCasNet: Two-stage detector+classifier: Cascade R-CNN + DiCoA (detect) → EfficientNet-B7 + VGG16 (re-classify).	Object-level detection	MITOS_CMC: F1 = 0.829; MITOS_CCMCT: F1 = 0.83
Shen (2024)	Multi-domain	In-house soft tissue tumor dataset (STMF), TUPAC16, MIDOG++, ICPR2012, MITOS_CMC, MITOS_CCMCT	MIDOG++	MIDOG++: Custom hold-out split	OMG-Net : Two-stage: SAM-based cell segmentation → ResNet18 classifier.	Object-level detection	F1 = 0.84
Tang (2024)	Breast	MIDOG21, ICPR2014	MIDOG21, ICPR2014	MIDOG21: CV, ICPR2014: CV	Two-stage: (1) candidate proposal FCN (classification + centroid-offset regression); (2) ResNet50 + RBF layer where RBF centers = spectral-clustering centroids, alternating cluster ↔ train.	Object-level detection	ICPR2014: F1 = 0.696; MIDOG21: F1 = 0.758
Taskeen (2024)	Breast	GZMH	GZMH	GZMH: Custom hold-out split	Two-stage instance segmentation / object detection: Mask R-CNN (ResNet-101 + FPN, RoIAlign), COCO-pretrained; ROI-based patch extraction + COCO JSON re-annotation from masks.	Object-level detection	F1 = 0.65
Lijo (2024)	Breast	ICPR2012	ICPR2012	ICPR2012: Official split/challenge test	Two-stage (segmentation → classification): stain normalization + enhancement + augmentation → U-Net segmentation (BCE + Dice loss) → modified VGG16 classifier on segmented ROIs.	Object-level detection	F1 = 0.80
Ding (2024)	Multi-domain	MIDOG22	MIDOG22	MIDOG22: Custom hold-out split	Vision–Language Models (VLMs): CLIP + BLIP formulated as image captioning and VQA (with metadata like tumor type / species / scanner).	Patch/cell-level classification	(BLIP VQA fine-tuned): F1 = 0.86
Wang (2024)	Breast	ICPR2012, GZMH	ICPR2012, GZMH	ICPR2012: Official split/challenge test, GZMH: Official split/challenge test	Two-stage cascaded detection + refinement: coarse detector Mdet (RetinaNet-style with Focal Loss) → classifier Mclass to remove hard negatives/FP; includes attention, normalization, improved ResNet feature re-encoding, and hybrid anchor branch for adaptive scale selection.	Object-level detection	ICPR2012 (FoCasNet): F1 = 0.88; GZMH (FoCasNet): F1 = 0.56
Topuz (2024)	Multi-domain	MIDOG22	MIDOG22, MIDOG++, -TUPAC16-Auxiliary, MITOS_CCMCT (MEL), ICPR2014	MIDOG22: Custom hold-out split, MIDOG++: External validation, TUPAC16-Auxiliary: External validation, MITOS_CCMCT: External validation, ICPR2014: External validation	Two-stage deep learning: YOLOv7 (cell detection) + ConvNeXt (cell classification).	Object-level detection	MIDOG22: F1 = 0.795; external melanoma (MIDG++): F1 = 0.783; external sarcoma (MIDG++): F1 = 0.759; ICPR2014: F1 = 0.49; TUPAC16-Auxiliary: F1 = 0.68; MITOS_CCMCT (MEL): F1 = 0.72
Shihabuddin (2024)	Breast	ICPR2014, TUPAC16	ICPR2014, TUPAC16	ICPR2014: Custom hold-out split, TUPAC16: Custom hold-out split	Single-stage patch pipeline: stain normalization → crop nuclei patches → multiCNN feature extraction (VGG16 + ResNet50 + DenseNet201)→ (optional PCA) → classical classifier (best reported: Linear SVM / LinearSVC)	Object-level detection	ICPR2014: F1 = 0.913; TUPAC16: F1 = 0.886
Alhassan (2025)	Multi-domain	MITOS_CCMCT, Mitosis-AIC, Mitosis Detection, Mitosis & Non-Mitosis	MITOS_CCMCT, Mitosis-AIC, Mitosis Detection, Mitosis & Non-Mitosis	MITOS_CCMCT: Custom hold-out split, Mitosis-AIC: Custom hold-out split, Mitosis Detection: Custom hold-out split, Mitosis & Non-Mitosis: Custom hold-out split	Customized Deep Learning (CDL): transfer learning + skip connections + hybrid feature selection / optimization (Jellyfish Search Optimizer + Walrus Optimization Algorithm).	Patch/cell-level classification	MITOS_CCMCT[7]: accuracy = 98.8%; Mitosis-AIC: accuracy = 98.5%; Mitosis Detection: accuracy = 98.3%; Mitosis & Non-Mitosis: accuracy = 98.1%
Arslanoglu (2025)	Multi-domain	MIDOG21, MIDOG22, ICPR2014	MIDOG21, MIDOG22, ICPR2014	MIDOG21: Custom hold-out split, MIDOG22: Custom hold-out split, ICPR2014: Custom hold-out split	Two-stage pipeline: (1) traditional segmentation (median filter + k-means + blob analysis) → candidate extraction; (2) classification with either supervised training (train backbone) or self-supervised DINO (frozen backbone + 2-layer linear classifier) using ViT/XCiT/ResNet-50 backbones.	Object-level detection	Best supervised F1: MIDOG21 0.8254, MIDOG22 0.8390, ICPR2014 0.7884; Best DINO F1: MIDOG21 0.8174, MIDOG22 0.8275, ICPR2014 0.7509

7 Dataset-level results are reported as accuracy for each dataset (Table 3 in their paper). Additional metrics including F-measure/F1 are presented (Table 2 in their paper), but the corresponding dataset/split is not explicitly stated; thus, F1 is not attributed to a specific dataset in our extraction and the study is treated as accuracy-only for cross-study comparisons. F1 in Table 2 numerically matches the dataset with 98.8% accuracy (CCMCT), but the mapping is not explicitly stated by the authors.

Study	Domain/ tissue	Train datasets	Test datasets	Evaluation type	Method category	Task type	Best reported result (test set)
Han (2025)	Multi-domain	MIDOG++ (MIDOG++ (6/7 domains), MIDOG21 (train for the external validation)	MIDOG++, MIDOG21, TUPAC16, AMIDA13, ICPR2014	MIDOG++ (held-out 1/7 domain): Explicit domain generalization protocol, MIDOG21 (held-out 1/3 scanner): Explicit domain generalization protocol, TUPAC16: External validation, AMIDA13: External validation, ICPR2014: External validation	DGDMD: One-stage anchor-free detector with dynamic depth-wise convolution backbone + domain alignment regularizers (GRL-style).	Object-level detection	MIDOG++: F1 = 0.763; MIDOG21 (scanner DG): F1 = 0.820; External (train MIDOG21 → test): TUPAC16 F1 = 0.700; AMIDA13 F1 = 0.688; ICPR2014 F1 = 0.507
Kanadath (2025)	Breast	GZMH	GZMH, MIDOG21	GZMH: Custom hold-out split, MIDOG21: External validation	HVUNet: Hybrid CNN–Transformer encoder–decoder (UNet + ViT): CMA encoder/decoder blocks, dual transformer bottleneck, decoder feature fusion; outputs localization/segmentation-style maps for small objects of interest.	Object-level detection	GZMH: F1 = 0.72; MIDOG21: F1 = 0.70
Kusuma Sri (2025)	Breast	ICPR2012, ICPR2014	ICPR2012, ICPR2014	ICPR2012: Official split/challenge test, ICPR2014: Custom hold-out split	QSLN-VTAN: ViT attention network + quadratic feature aggregation/classifier + contrast/luminance-enhanced stain normalization.	Patch/cell-level classification	ICPR2012: F1 = 0.93; ICPR2014: F1 = 0.91
Li (2025)	Multi-domain	MIDOG22	MIDOG22	MIDOG22: Custom hold-out split	One-stage object detection: YOLO11-L baseline + Block-Based Mixed Mechanism (BBMM) as backbone + hypergraph convolution (HGNN) in neck + bottom-up refinement head.	Object-level detection	F1 = 0.863
Nemati (2025b)	Breast	MIDOG21, TUPAC16, MiDeSeC	MIDOG21; TUPAC16; MiDeSeC	MIDOG21: Custom hold-out split, TUPAC16: Custom hold-out split, MiDeSeC: Custom hold-out split	HR-YOLOv8: YOLOv8 + HRNet (HR-YOLOv8) with HSV + wavelet preprocessing.	Object-level detection	MiDeSeC: F1 = 0.912; TUPAC16: F1 = 0.922; MIDOG21: F1 = 0.94

CV: Cross validation
