# Supplementary material for: Recent Advances in Automated Mitosis Detection in Digital Pathology: A PRISMA-Guided Systematic Review with Evaluation-Regime Stratification (2018–2025)
Source: Biomedicines. 2026 Jun 17;14(6):1369. doi: 10.3390/biomedicines14061369 (PMC13296622; doi:10.3390/biomedicines14061369)
Supplement: Supplementary file 1 [file biomedicines-14-01369-s001.zip › biomedicines-4334488-supplementary/Supplementary Files/Supplementary Data S2.pdf]

Supplementary Data B: Extraction Table for the public datasets and challenge benchmarks used for training and evaluation of mitosis detection in this review (2018–2025)

Dataset / benchmark (release)	Primary domain (species / tissue)	Image context	Scale & size (as reported)	Annotation type	Typical use in literature	Availability
ICPR2012	Human breast cancer	HPF images (multispectral; z-stack)	50 HPFs (5 slides), 322 mitoses; train: 35 HPFs (224), test/eval: 15 HPFs (98)	Mitosis locations/regions (challenge annotations)	Legacy benchmark for HPF/patch pipelines; controlled comparisons	Public
ICPR 2014	Human breast cancer	HPF/ROI images (paired scanners)	Train: 1200 Aperio XT + 1200 Hamamatsu; Test: 496 Aperio XT + 496 Hamamatsu (paired)	Point/centroid mitosis annotations (challenge)	Patch/ROI benchmark; stain/scanner robustness comparisons	Train: Public; Test: Not public
AMIDA13	Human breast cancer (multi-center)	HPF/ROI images (challenge)	Train: 12 subjects; Test: 11 subjects	Point/centroid mitosis annotations (challenge)	WSI/center variability benchmark; cross-center generalization	Public
TUPAC16	Human breast cancer	WSI + auxiliary mitosis detection ROIs	73 cases	Point/centroid mitosis annotations on selected ROIs	Cross-dataset generalization; robustness testing	Public
MIDOG21	Human breast cancer (multi-scanner)	ROI images (challenge format)	Training: 200 cases; Test includes unlabeled domain(s) with hidden labels	Point/centroid mitosis annotations (challenge)	Scanner domain-generalization benchmark	Train: Public; Test: Not public
MIDOG22	Multi-domain (tumor type / lab / scanner / species)	ROI images (challenge format)	Training: 405 cases; Test includes unlabeled domain(s) with hidden labels	Point annotations (mitoses) + hard negatives (challenge ground truth)	Strong multi-domain DG benchmark; detector and detector→classifier pipelines	Train: Public; Test: Not public
MIDOG++	Multi-domain (human + canine; multiple tumor types)	ROI images (standardized ROIs)	503 cases	Point/centroid mitosis annotations across domains	Consolidated multi-domain benchmark for robustness/DG analyses	Public (open dataset release)
MiDeSeC	Human breast cancer	ROI images (HPF-like regions)	25 patients, 50 regions	Pixel-level mitosis masks (per-mitosis pixels)	Joint detection + segmentation evaluation (explicit mask/shape handling)	Public

GZMH dataset (Ganzhou Municipal Hospital; breast)	Human breast cancer	HPF images (+ WSI mentioned)	HPF: 1192 train + 342 test; also mentions 55 WSIs (22 patients)	Detection annotations incl. bbox + centroid (text files)	Detector-style pipelines; sometimes paired with older breast benchmarks	Unclear
MITOS_CMC	Canine mammary carcinoma	Whole slide images (WSI)	21 WSIs	Dense WSI-scale annotations (mitoses + hard negatives)	Cross-species/domain shift; WSI-scale training/evaluation	Public
MITOS_CCMCT	Canine cutaneous mast cell tumor	Whole slide images (WSI)	32 WSIs	Dense WSI-scale annotations (mitoses + additional classes)	Veterinary morphology; hard-negative mining; WSI-scale evaluation	Public
CWRU (Case Western Reserve Univ.)	Human breast cancer	HPF images / patches	Training: 254 HPFs; Testing: 25 HPFs	Patch masks / patch labels (paper-dependent)	Auxiliary/private external validation in some studies	Restricted/unclear (not a standard public benchmark)
KMIT (private WSI dataset; "CADD4MBC" line of work)	Human breast cancer	Whole slide images (WSI → tiled)	Private; counts/splits reported per paper	Point/coordinate annotations on WSI tiles	Large private WSI training/testing to complement public sets	Private (not publicly released)
RCC clinical HPF dataset	Clinical H&E HPFs (breast in that work)	Microscope/HPF images	Not standardized public release; reported per paper	Mitotic vs non-mitotic patch labels	Clinical external validation alongside public ICPR folders	Private/clinical (not publicly released)
Kaggle: "Mitosis-AIC"	Patch classification	Image patches	Varies by Kaggle version / paper usage	Image-level labels (mitosis vs non-mitosis)	Multi-dataset patch classification papers	Public (Kaggle)
Kaggle: "Mitosis Detection"	Patch classification	Image patches	Varies by Kaggle version / paper usage	Image-level labels (mitosis vs non-mitosis)	Multi-dataset patch classification papers	Public (Kaggle)
Kaggle: "Mitosis and Non-Mitosis"	Patch classification	Image patches	Varies by Kaggle version / paper usage	Image-level labels (mitosis vs non-mitosis)	Multi-dataset patch classification papers	Public (Kaggle)
In-house (Çayır 2022 / "MITNET")	Human breast cancer	WSI patches + extracted mitosis/non-mitosis samples	Paper-defined (not a standardized benchmark)	Manual annotations (nuclei + mitosis/non-mitosis samples)	In-house training/testing; augmentation-heavy pipelines	Private (institutional dataset)
STMF	Human Soft tissue tumour	Image patches	938	Annotations created via pHH3 Detection, active learning	In-house training	Private (institutional dataset)
