# Supplementary material for: Recent Advances in Automated Mitosis Detection in Digital Pathology: A PRISMA-Guided Systematic Review with Evaluation-Regime Stratification (2018–2025)
Source: Biomedicines. 2026 Jun 17;14(6):1369. doi: 10.3390/biomedicines14061369 (PMC13296622; doi:10.3390/biomedicines14061369)
Supplement: Supplementary file 1 [file biomedicines-14-01369-s001.zip › biomedicines-4334488-supplementary/Supplementary Files/Supplementary Data S3.pdf]

Supplementary Data C: Search Queries

Database	Query	Number of resulted papers
PubMed	("mitotic figure"[Title/Abstract] OR "mitotic figures"[Title/Abstract] OR mitosis[Title/Abstract] OR mitoses[Title/Abstract]) AND (detect*[Title/Abstract] OR detection[Title/Abstract] OR localiz*[Title/Abstract] OR localis*[Title/Abstract] OR count*[Title/Abstract]) AND ("digital pathology"[Title/Abstract] OR histopathology[Title/Abstract] OR histology[Title/Abstract] OR "whole slide image"[Title/Abstract] OR "whole slide images"[Title/Abstract] OR "whole-slide image"[Title/Abstract] OR "whole-slide images"[Title/Abstract] OR WSI[Title/Abstract] OR WSIs[Title/Abstract] OR "haematoxylin and eosin"[Title/Abstract] OR "hematoxylin and eosin"[Title/Abstract] OR haematoxylin[Title/Abstract] OR hematoxylin[Title/Abstract] OR eosin[Title/Abstract] OR "H&E"[Title/Abstract] OR "H and E"[Title/Abstract]) AND ("deep learning"[Title/Abstract] OR "machine learning"[Title/Abstract] OR "artificial intelligence"[Title/Abstract] OR "convolutional neural network"[Title/Abstract] OR CNN[Title/Abstract] OR "patch-based"[Title/Abstract] OR "patch classification"[Title/Abstract] OR "two-stage"[Title/Abstract] OR "multi-stage"[Title/Abstract] OR "weakly supervised"[Title/Abstract] OR pipeline*[Title/Abstract] OR approach*[Title/Abstract] OR dataset*[Title/Abstract] OR benchmark*[Title/Abstract] OR challenge*[Title/Abstract] OR MIDOG[Title/Abstract] OR TUPAC[Title/Abstract] OR MITOS[Title/Abstract] OR "domain shift"[Title/Abstract] OR "domain generalization"[Title/Abstract] OR "foundation model"[Title/Abstract] OR "foundation models"[Title/Abstract] OR "Segment Anything"[Title/Abstract] OR SAM[Title/Abstract] OR PathoSAM[Title/Abstract])	107
Scopus	TITLE-ABS-KEY (("mitotic figure" OR "mitotic figures" OR mitosis OR mitoses) AND (detect* OR detection OR localiz* OR localis* OR count*) AND ("digital pathology" OR histopathology OR histology OR "whole slide image" OR "whole slide images" OR "whole-slide image" OR "whole-slide images" OR WSI OR WSIs OR "haematoxylin and eosin" OR "hematoxylin and eosin" OR haematoxylin OR hematoxylin OR eosin OR "H&E" OR "H and E") AND ("deep learning" OR "machine learning" OR "artificial intelligence" OR "convolutional neural network" OR CNN OR "patch-based" OR "patch classification" OR "two-stage" OR "multi-stage" OR "weakly supervised" OR pipeline* OR approach* OR dataset* OR benchmark* OR challenge* OR MIDOG OR TUPAC OR MITOS OR "domain shift" OR "domain generalization" OR "foundation model" OR "foundation models" OR "Segment Anything" OR SAM OR PathoSAM)) AND PUBYEAR > 2017 AND PUBYEAR < 2026 AND (LIMIT-TO (DOCTYPE , "ar")) AND (LIMIT-TO (LANGUAGE , "English"))	341
IEEE Xplore	((("mitotic figure" OR "mitotic figures" OR mitosis OR mitoses) AND (detect* OR detection OR localization OR localisation OR count*) AND ("digital pathology" OR histopathology OR histology OR "whole slide image" OR "whole slide images" OR "whole-slide image" OR "whole-slide images" OR WSI OR WSIs OR "haematoxylin and eosin" OR "hematoxylin and eosin" OR haematoxylin OR hematoxylin OR eosin OR "H&E" OR "H and E") AND ("deep learning" OR "machine learning" OR "artificial intelligence" OR "convolutional neural network" OR CNN OR "patch-based" OR "patch classification" OR "two-stage" OR "multi-stage" OR "weakly supervised" OR pipeline* OR approach* OR dataset* OR benchmark* OR challenge* OR MIDOG OR TUPAC OR MITOS OR "domain shift" OR "domain generalization" OR "foundation model" OR "foundation models" OR "Segment Anything" OR SAM OR PathoSAM)))	51
