# Supplementary material for: Recent Advances in Automated Mitosis Detection in Digital Pathology: A PRISMA-Guided Systematic Review with Evaluation-Regime Stratification (2018–2025)
Source: Biomedicines. 2026 Jun 17;14(6):1369. doi: 10.3390/biomedicines14061369 (PMC13296622; doi:10.3390/biomedicines14061369)
Supplement: Supplementary file 1 [file biomedicines-14-01369-s001.zip › biomedicines-4334488-supplementary/Supplementary Files/Supplementary Data S4.pdf]

Supplementary Data D: Quality assessment methods

Modified QUADAS-2 definitions and signalling questions

This supplementary file documents the operational definitions and signalling questions used to assess risk of bias and applicability for algorithm studies on automated mitotic figure detection in H&E-stained digital pathology.

The instrument is an adaptation of QUADAS-2 (Whiting *et al.*, 2011) to reflect common study designs in computational pathology (dataset provenance, split strategy, annotation standards, and evaluation protocols).

Response options for signalling questions: Yes / No / Unclear.

1. Definitions

Concept	Operational definition for this review
**Population (participants/data)**	Histopathology images used to develop and evaluate the algorithm (whole-slide images or patches), including provenance (public benchmark vs. private/in-house), tissue type, staining, scanner and laboratory context.
**Reference standard**	Ground truth mitosis labels derived from expert annotation (single or multiple pathologists, with or without consensus). For public challenges, this refers to organizer-provided annotations and evaluation servers.
**Index test**	The automated mitotic figure detection algorithm under evaluation (classical ML or deep learning; detection/segmentation/cascade formulations), including its training and model selection process.
**Outcomes and analysis**	Evaluation protocol and metrics used to compare algorithm outputs with the reference standard (e.g., F1, precision/recall, average precision), plus reporting of exclusions and variability/uncertainty where applicable.
**Applicability**	Extent to which data, task definition, and outcomes match the intended deployment setting (target tissues/tumors, clinical workflow, scanner/lab diversity, and clinically meaningful endpoints).

2. Risk of bias domains (modified QUADAS-2)

Domain 1: Participants (data selection and split integrity) – Risk of bias

Signalling question	Guidance for **Yes / No / Unclear**
**Q1. Split unit appropriate (patient/WSI-level separation)**	Yes — Split is defined at an independent unit (e.g., patient or whole slide) and prevents overlap.No — Split is at patch/tile level only, or overlap across sets is likely/confirmed.Unclear — Split unit is not reported or cannot be inferred.
**Q2. No information leakage stated**	Yes — Test data were not used for model selection; leakage prevention is explicitly stated.No — Test-informed tuning/selection or leakage is reported/strongly suggested.Unclear — Leakage prevention is not described.
**Q3. Selection and sampling described**	Yes — Inclusion/exclusion criteria, sampling strategy, and selection-related pre-processing are described.No — Selection is evidently restrictive/biased without justification, or key exclusions are undocumented.Unclear — Insufficient detail on how cases/images were selected.
**Domain judgement (D1)**	Low — Q1=Yes AND Q2=Yes AND Q3=Yes.High — Q1=No OR Q2=No.Unclear — All other combinations.

Domain 2: Index test (algorithm development and evaluation integrity) – Risk of bias

Signalling question	Guidance for **Yes / No / Unclear**
**Q4. Model selection performed using**	Yes — Hyper-parameters/early stopping/model selection are based on training/validation only.No — The test set is used for tuning/iterative selection.Unclear — Model selection

Signalling question	Guidance for **Yes / No / Unclear**
**validation data only**	process is not described.
**Q5. Decision threshold fixed before test evaluation**	Yes — Threshold(s) and post-processing are pre-specified or fixed using validation only.No — Threshold(s) are chosen based on test performance.Unclear — Threshold selection is not reported.
**Q6. Blinded evaluation w.r.t. reference standard**	Yes — Test evaluation is conducted without access to test labels (e.g., locked test set, challenge server, or clearly separated evaluation).No — Test labels are accessible during development in a way that could bias evaluation.Unclear — Blinding/label access is not described.
**Domain judgement (D2)**	Low — Q4=Yes AND Q5=Yes AND Q6=Yes.High — Any of Q4–Q6 is No.Unclear — All other combinations.

Domain 3: Reference standard (mitosis annotation quality) – Risk of bias

Signalling question	Guidance for **Yes / No / Unclear**
**Q7. Annotation process described**	Yes — Annotation workflow is described (who annotated, how, format, quality control).No — Reference standard is inadequately described or clearly unreliable.Unclear — Only minimal mention of "ground truth" without methodology.
**Q8. Multiple annotators and/or consensus used (or justified)**	Yes — Multi-annotator labelling/consensus/adjudication, or a justification for single-expert labelling is provided.No — Single-annotator labelling without justification likely to introduce bias/label noise.Unclear — Annotator information not reported.
**Q9. Label definition and annotation target are clear**	Yes — Operational definition of mitosis and label granularity (point/box/mask) are defined.No — Label definition is ambiguous/inconsistent or incompatible with evaluation.Unclear — Label definition not stated.
**Domain judgement (D3)**	Low — Q7=Yes AND Q8=Yes AND Q9=Yes.High — Q7=No OR Q9=No.Unclear — All other combinations.

Domain 4: Outcomes and analysis (reporting completeness and protocol governance) – Risk of bias

Signalling question	Guidance for **Yes / No / Unclear**
**Q10. Same reference standard applied to all evaluated images**	Yes — Ground truth is consistently applied across all evaluated images. No — Different/inconsistent reference standards are used within the evaluated set.Unclear — Consistency not described.
**Q11. Exclusions and missing data are reported**	Yes — Exclusions and missing annotations/data are reported and justified. No — Selective exclusion is evident/likely without reporting.Unclear — Insufficient reporting to determine exclusions.
**Q12. Challenge server / official test evaluation confirmed (when claimed)**	Yes — For official/challenge claims, submission to an organizer-governed server/test is confirmed. No — Official/challenge is claimed but evidence is inconsistent with a governed protocol.Unclear — Cannot be verified.
**Domain judgement (D4)**	Low — Q10=Yes AND Q11=Yes AND (if official/challenge claimed: Q12=Yes).High — Q10=No OR Q11=No OR (official/challenge claimed AND Q12=No).Unclear — All other combinations.

Overall risk-of-bias judgement

Overall risk of bias is computed as a worst-case judgement across Domains 1–4:

- Low only if all domains are Low
- High if any domain is High
- Unclear otherwise

3. Applicability concerns (review-specific extension)

Applicability was assessed using three signalling questions tailored to mitosis detection in digital pathology. Each item is judged Low / Unclear / High; the overall applicability judgement follows a worst-case rule (High if any item is High; Low if all items are Low; otherwise Unclear).

Signalling question	Guidance for **Yes / No / Unclear**
**A1. Target setting matches intended deployment setting (Participant applicability)**	Yes — Test data represent the intended clinical setting (relevant tissue/tumor context; realistic staining/scanner conditions).No — Data are from a clearly mismatched setting without justification.Unclear — Target setting/provenance insufficiently described.
**A2. Task definition maps to deployment needs (Index test applicability)**	Yes — Evaluated task (label granularity, regions, counting protocol) aligns with grading workflows.No — Task definition is not clinically actionable or mismatched to intended use.Unclear — Insufficient detail to determine alignment.
**A3. Outcome is clinically meaningful and reported transparently (Outcome applicability)**	Yes — Primary metric(s) and operating point(s) are appropriate and clearly reported.No — Outcomes are not clinically meaningful or reporting prevents interpretation.Unclear — Outcomes/operating points not sufficiently specified.
**Applicability judgement (overall)**	Low — A1=Yes AND A2=Yes AND A3=Yes.High — Any of A1–A3 is No.Unclear — All other combinations.
