# Supplementary material for: Recent Advances in Automated Mitosis Detection in Digital Pathology: A PRISMA-Guided Systematic Review with Evaluation-Regime Stratification (2018–2025)
Source: Biomedicines. 2026 Jun 17;14(6):1369. doi: 10.3390/biomedicines14061369 (PMC13296622; doi:10.3390/biomedicines14061369)
Supplement: Supplementary file 1 [file biomedicines-14-01369-s001.zip › biomedicines-4334488-supplementary/Supplementary Files/Supplementary Data S5.pdf]

1. Supplementary Methods S1. Evaluation-regime classification rules

Evaluation regimes were coded according to the evaluation setting explicitly linked to the reported results in each study. The following categories were used: official split/challenge test, custom hold-out split, cross-validation, external validation, explicit domain generalization, and unclear/insufficiently specified.

Official split/challenge test was assigned when the study reported evaluation using organizer-defined benchmark partitions, challenge protocols, hidden-label test sets, or official scoring procedures. Custom hold-out split was assigned when authors defined their own train/test split within a dataset or across pooled datasets and the split was not described as an official organizer-defined benchmark partition. Cross-validation was assigned to k-fold or repeated resampling evaluation. External validation was assigned when a model was trained on one named dataset family and tested on a different named dataset family. Explicit domain generalization was assigned when one or more domains, such as scanner, laboratory, tissue/domain, or dataset domain, were held out from training by design and used only for testing. Unclear/insufficiently specified was assigned when the publication did not provide enough information to determine the evaluation regime without inference.

Where studies reported multiple evaluation regimes, each regime was preserved in the evidence table rather than collapsed into a single study-level label.

2. Supplementary Methods S2: Method-family decision rules

Each algorithmic study was assigned to one primary method family based on the dominant localization mechanism used to generate candidate mitosis locations. This rule was used because many studies used hybrid designs, such as detector-to-classifier, segmentation-to-classifier, or candidate-generator-to-refinement pipelines. When multiple components were present, the primary family reflected the component responsible for the main localization step rather than downstream verification alone.

The following primary method families were used:

Two-stage region/proposal detectors. Studies were assigned to this family when mitosis localization was driven by a proposal-based detection architecture, such as Faster R-CNN-, Mask R-CNN-, or Cascade R-CNN-style systems, including cases with downstream screening or refinement.

One-stage object detectors. Studies were assigned to this family when localization was performed by a single-pass detector without an explicit proposal stage, including YOLO-, RetinaNet-, DETR-style, anchor-free, or related detector formulations.

Dense segmentation or heatmap-based detection. Studies were assigned to this family when mitosis localization was formulated as dense prediction, semantic segmentation, pixel-wise scoring, probability-map prediction, or heatmap regression followed by centroid, peak, or connected-component extraction.

Candidate-based cascades. Studies were assigned to this family when the pipeline explicitly separated high-recall candidate generation from subsequent classification or false-positive suppression, and the candidate-generation stage remained central to localization.

Handcrafted/classical machine-learning pipelines. Studies were assigned to this family when the main discriminative mechanism relied primarily on engineered features, such as morphology, texture, intensity, or color descriptors, combined with classical classifiers or ensembles.

Deep feature extraction or patch/cell-level classification. Studies were assigned to this family when the main inferential unit was a patch, cell, or candidate crop classified using deep features or CNN-based classification, rather than direct object detection.

Representation learning/domain adaptation/domain generalization. Studies were assigned to this family when the main methodological contribution emphasized representation robustness, unsupervised domain adaptation, explicit domain generalization, self-supervised learning, or contrastive pretraining, even if the final downstream task used a detector or dense prediction model.
